# Mitochondrial transfer from cancer-associated fibroblasts increases migration in aggressive breast cancer

**DOI:** 10.1242/jcs.260419

**Published:** 2023-07-28

**Authors:** Kayla F. Goliwas, Sarah Libring, Emily Berestesky, Shayan Gholizadeh, Samantha C. Schwager, Andra R. Frost, Thomas R. Gaborski, Jian Zhang, Cynthia A. Reinhart-King

**Affiliations:** ^1^ Department of Biomedical Engineering, Vanderbilt University, Nashville, TN 37235, USA; ^2^ Department of Biomedical Engineering, Rochester Institute of Technology, Rochester, NY 14623, USA; ^3^ Department of Pathology, University of Alabama at Birmingham, Birmingham, AL 35233, USA; ^4^ Department of Biomedical Engineering, University of Arkansas, Fayetteville, AR 72701, USA

**Keywords:** Reverse Warburg effect, CAF, Tunneling nanotube, Tumor microenvironment, Bioenergetics, Tumor spheroid, ATP production

## Abstract

Cancer-associated fibroblasts (CAFs) have distinct roles within the tumor microenvironment, which can impact the mode and efficacy of tumor cell migration. CAFs are known to increase invasion of less-aggressive breast cancer cells through matrix remodeling and leader–follower dynamics. Here, we demonstrate that CAFs communicate with breast cancer cells through the formation of contact-dependent tunneling nanotubes (TNTs), which allow for the exchange of cargo between cell types. CAF mitochondria are an integral cargo component and are sufficient to increase the 3D migration of cancer cells. This cargo transfer results in an increase in mitochondrial ATP production in cancer cells, whereas it has a negligible impact on glycolytic ATP production. Manually increasing mitochondrial oxidative phosphorylation (OXPHOS) by providing extra substrates for OXPHOS fails to enhance cancer cell migration unless glycolysis is maintained at a constant level. Together, these data indicate that tumor–stromal cell crosstalk via TNTs and the associated metabolic symbiosis is a finely controlled mechanism by which tumor cells co-opt their microenvironment to promote cancer progression and may become a potential therapeutic target.

## Teaser

**Highlighted Article:** Cancer-associated fibroblasts impart a migratory advantage onto already highly invasive breast cancer cell spheroids owing, in part, to mitochondrial transfer via tunneling nanotubes and the associated bioenergetic rewiring.

## INTRODUCTION

The tumor microenvironment, including not only physical and chemical cues imposed by the extracellular matrix (ECM) but also resident stromal cell populations, can influence many facets of tumor biology. This includes the mode and dynamics of cancer cell migration, which are critical processes within the metastatic cascade for tumor cell dissemination and the generation of metastatic lesions at secondary sites (Gligorijevic et al., 2014; Polacheck et al., 2013). Cancer-associated fibroblasts (CAFs) are the predominant multi-functional stromal cells involved in the deposition of ECM and secretion of growth factors that can support (Dumont et al., 2013; van Zijl et al., 2009) or restrict (Gieniec et al., 2019; Rhim et al., 2014; Ozdemir et al., 2014) tumor growth and metastasis.

Collective migration, where groups of cells retaining cell–cell contacts invade together, is a strategy utilized by many cancers during invasion and metastasis (Cheung and Ewald, 2016). Often, cells of a more invasive phenotype, such as CAFs or highly invasive cancer cells, lead dissemination from the tumor for non- or less-invasive follower cells (Carey et al., 2013). CAFs, as leader cells, have been associated with cooperative migration through their roles in matrix remodeling – such as by altering the alignment of fibronectin fibrils (Attieh et al., 2017) or by degrading the ECM to form microtracks (Gaggioli et al., 2007) – and through their role in physical force transmission (Labernadie et al., 2017). Although CAFs are known to increase invasion of less-aggressive breast cancer cells, often through these leader–follower dynamics, the role of CAFs in migration of more aggressive breast cancer cells remains unclear. Additionally, CAFs have recently been shown to support tumor growth and dissemination through other methods as well, such as by altering the metabolic profile of cancer cells, and their roles in the invasion of aggressive breast cancers was also unknown (Liang et al., 2022; Liu et al., 2019).

Among others, two prominent mechanisms through which stromal cells can alter cancer cell metabolism are the reverse Warburg effect and through transferring mitochondrial cargo. The Warburg effect is a well-studied phenomenon where cancer cells favor glycolysis over OXPHOS even in aerobic conditions, resulting in increased glucose uptake and upregulation of glucose transporters (Zanotelli et al., 2021). However, cancer cells can also induce aerobic glycolysis in surrounding stromal cells and use waste metabolites from those cells, such as lactate and pyruvate, to undergo additional OXPHOS reactions (Liang et al., 2022). This is known as the reverse Warburg effect. In this way, cancer cells can increase both glycolytic and mitochondrial ATP levels beyond non-transformed cells through the Warburg effect and the reverse Warburg effect, respectively, depending on the changing demands of the cancer cells and their microenvironment (Fu et al., 2017).

Mitochondrial transfer is described as the movement of whole mitochondria or mitochondrial genes from a host to a recipient cell. This transfer can occur through several means, but the formation of tunneling nanotubes (TNTs) is increasingly recognized as the main mechanism (Plotnikov et al., 2015). The transported material has been reported to impart chemoresistance, increase OXPHOS and increase cancer cell proliferation, depending on the cell involved and the experimental parameters (Zampieri et al., 2021). However, although there are an increasing number of studies on mitochondrial transfer in cancer (Burt et al., 2019; Ippolito et al., 2019; Lu et al., 2017; Saha et al., 2022), information is still limited and the effects on both recipient cell metabolism and cancer progression warrant further investigation.

Given the various reported tumor-promoting and tumor-restricting contributions of CAFs, a gap remains in the field to identify the role of CAFs in aggressive breast cancer tumors, especially when the cancer cells could lead invasion independently. Within tumor-promoting roles, it was particularly unknown whether CAFs would support invasion primarily through leader–follower dynamics and matrix remodeling, lactate and pyruvate secretion, or mitochondrial transfer. Using a three-dimensional (3D) spheroid model to co-culture highly invasive human breast epithelial cancer cells (MDA-MB-231 and SUM159) with patient-derived CAFs, we demonstrated that cancer cell invasion is enhanced by CAF co-cultures, but this is not primarily the result of CAFs functioning as leader cells. Instead, we found that tumor cells acquired mitochondria-containing cargo from CAFs via contact-dependent TNT formation. ‘Pre-education’ of breast cancer cells with CAFs via two-dimensional (2D) co-culturing, as well as artificial transfer of CAF mitochondria to cancer cells, was sufficient to increase migration in tumor-only spheroids. We further found that this pre-education increased mitochondrial ATP production rate without affecting glycolytic production rate. However, we demonstrated that the addition of pyruvate is not sufficient to increase, and instead inhibits, invasion in highly aggressive breast cancer cells, indicating that the CAF co-culturing, which enables the transfer of exogenous mitochondria to cancer cells, has effects beyond those traditionally described in the reverse Warburg effect.

## RESULTS

### CAFs within spheroids promote cancer cell migration

**Fig. 1.**
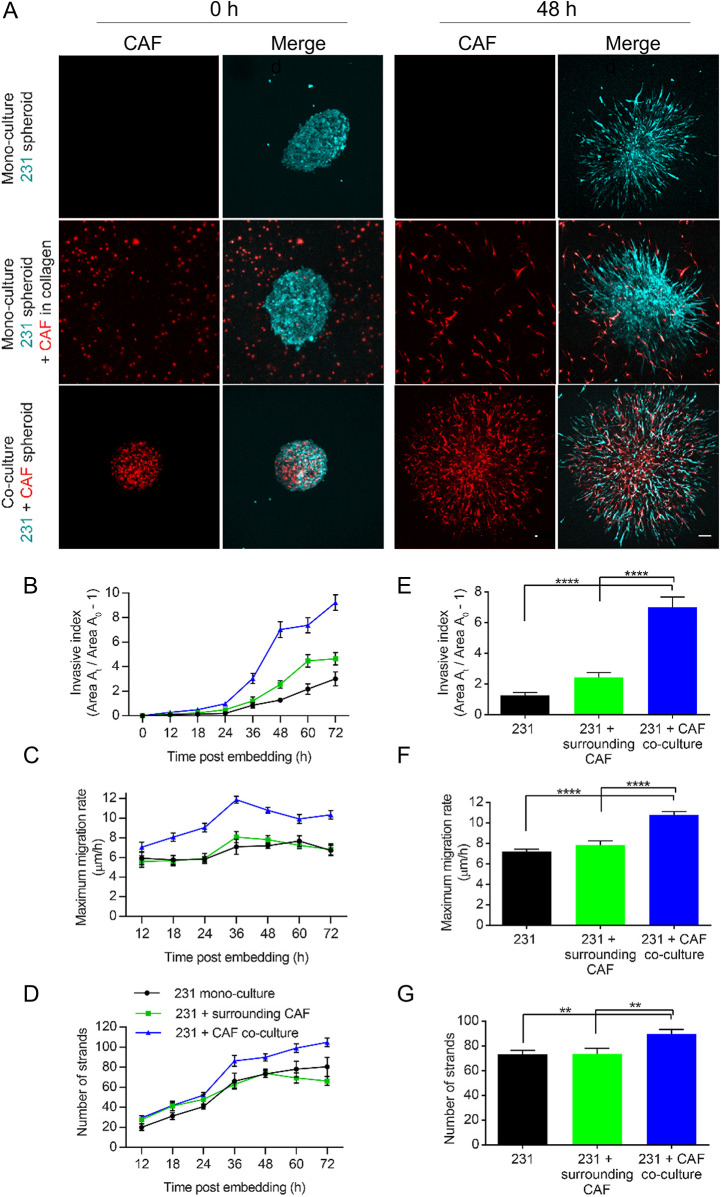
**CAFs within spheroids promote cancer cell migration.** (A) Representative images of a mono-culture CellTracker-labeled MDA-MB-231 (cyan) spheroid alone (top) or with CAF01-mCherry (red) in the surrounding collagen matrix (middle), and a co-culture spheroid (CellTracker-labeled MDA-MB-231 and CAF01-mCherry at 2:1 ratio; bottom) at 0 h and 48 h post embedding in 4.5 mg/ml collagen matrices. Scale bar: 100 µm. (B) Invasive index, (C) maximum migration rate and (D) number of strand protrusions of mono-culture spheroids with or without surrounding CAF01-mCherry, and co-culture spheroids over 3 days. (E) Co-culture spheroids (*n*=27) have significantly increased invasive index at 48 h post embedding, as compared to mono-culture spheroids with (*n*=17) or without (*n*=22) surrounding CAF01-mCherry. Co-culture spheroids (*n*=28) have significantly increased maximum migration rate (F) and number of strand protrusions (G) at 48 h post embedding, as compared to mono-culture spheroids with (*n*=16) or without (*n*=19) surrounding CAF01-mCherry. Data pooled from a minimum of three independent experiments. Error bars are mean±s.e.m. **P<0.01, ****P<0.0001 [one-way ANOVA followed by Sidak's multiple comparison testing (E–G)].

We first established the role of CAF01 cells, isolated from remnant human breast cancer tissue, on the migration of MDA-MB-231 human breast cancer cells away from a tumor spheroid in type I rat-tail collagen. We compared migration of breast cancer spheroids without any CAF intervention, a breast cancer cell spheroid surrounded by CAFs in the collagen network, and a heterogeneous mix of cancer and stromal cells in the spheroid. Tumor spheroids were composed of 5000 total cells as either mono-culture spheroids of MDA-MB-231 cells (Zhang et al., 2019) or co-culture spheroids of MDA-MB-231 and CAF01–mCherry cells at a 2:1 ratio (Labernadie et al., 2017). After allowing the cells to compact in round-bottom plates for 3 days, the spheroids were embedded in 4.5 mg/ml collagen with or without the presence of surrounding CAF01–mCherry cells in the matrix (Fig. 1A) (Attieh et al., 2017). To quantify migration dynamics, the invasive index, the maximum migration rate, and the number of strand protrusions were evaluated over time. The invasive index is a normalized measurement of spheroid cross-sectional area (see Materials and Methods for details), whereas maximum migration rate is based on the leader cell that migrated farthest away from the spheroid. Migration from the spheroid was observed in all conditions (Fig. 1A), with invasive index and number of strand protrusions generally increasing across three days of analysis, and the maximum migration rate staying relatively consistent in each condition (Fig. 1B–D). However, co-culture spheroids showed an increased invasive index, maximum migration rate and number of strand protrusions compared to mono-culture spheroids with or without CAFs in the surrounding collagen. Here and in subsequent experiments, analysis was performed at 48 h post-embedding in collagen, a time at which heterogeneous co-culture spheroids show significant increase in these three migration metrics over mono-culture spheroids with or without surrounding CAFs (Fig. 1E–G). Similar results were confirmed using MDA-MB-231 cells with CAFs derived from a second patient (CAF32) (Fig. S1A) and using SUM-159 human breast cancer cells with the CAF01 cells (Fig. S1B–D).

### CAFs transfer cargo to breast cancer cells through TNTs

**Fig. 2.**
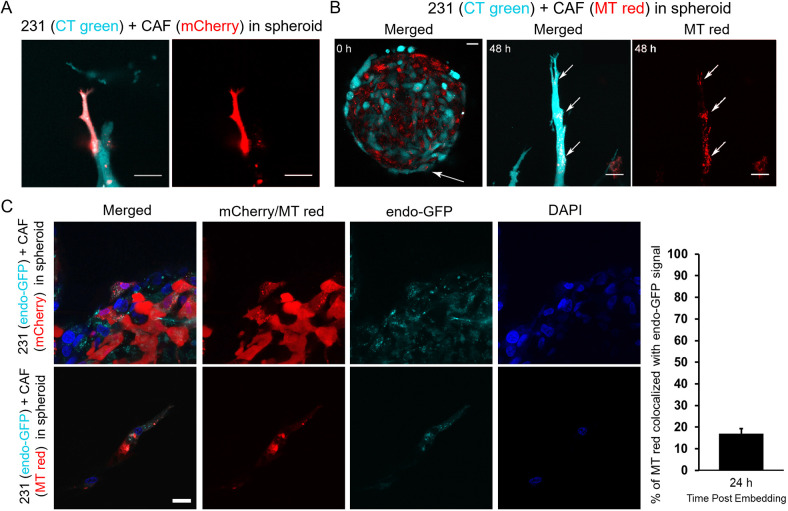
**CAFs transfer mitochondria-containing cargo to breast cancer cells.** (A) Representative image of CAF01-mCherry-led strand protrusion 48 h post embedding from a co-culture spheroid in 4.5 mg/ml collagen. CAF-derived mCherry signal (red) can be seen localized within the CellTracker (CT Green)-labeled MDA-MB-231 cell (cyan). Scale bars: 20 µm. (B) Representative images of a co-culture spheroid of CT Green-labeled MDA-MB-231 (cyan) and MitoTracker Red (MT Red)-labeled CAF01 (red) cells within the collagen matrix at 0 h and an invading strand at 48 h post embedding, showing the transfer of mitochondria from CAFs to cancer cells (as indicated by white arrows). Scale bars: 20 µm. (C) Representative images of co-culture spheroid containing MDA-MB-231-endosome-GFP (cyan) and CAF01-mCherry (top; red) or MT Red-labeled CAF01 (bottom; red) within the collagen matrix at 24 h post embedding, with cell nuclei stained with DAPI (blue). Scale bar: 20 µm. Image analysis of cells from six independent 40× objective images (right) indicates that 17.1±2.2% (mean±s.e.m.) of MT Red pixels within MDA-MB-231 cells were colocalized with the endosome signal. All images representative of results from a minimum of three experiments.

Because increased invasion was not significantly enhanced by CAFs in the surrounding matrix, we hypothesized that contact-dependent communication between CAFs and cancer cells was necessary for the migratory advantage seen. Consistent with this hypothesis, punctate regions of CAF-derived mCherry were observed within MDA-MB-231 cells in co-culture spheroid experiments (Fig. 2A; Movie 1), indicating possible cargo transfer from CAFs to breast cancer cells. Additionally, with respect to the location of the CAFs in the collagen, we saw significantly fewer CAF-led strands from the heterogeneous co-culture spheroids than from mono-culture spheroids surrounded by CAFs, and in both experimental setups, CAF-led strands migrated a smaller distance by 48 h than corresponding cancer-lead strands (Fig. S2A,B). Using confocal reflectance (Fig. S2C), we also noted that although the architecture of the collagen matrix did become more aligned over time, no differences were observed between mono- and co-culture spheroids at 0, 12 or 48 h post embedding as a function of CAF placement (Fig. S2D). These data indicate that the increase in migration observed in co-culture spheroids is not due to traditional leader–follower dynamics or changes in the collagen architecture.

We next sought to investigate the cargo and the method of cargo transfer observed between the cell types. Cellular cargo transfer can occur through a variety of methods, including extracellular vesicle (EV) release and uptake (Mulvey et al., 2015; Schwager et al., 2019; Steenbeek et al., 2018) and contact-dependent transfer via TNTs (Burt et al., 2019; Lu et al., 2017; Gerdes and Carvalho, 2008; Rustom et al., 2004). To investigate the transfer method and cargo contents, we utilized dyes for mitochondria and endosomes in two experiments. First, to identify the cargo, heterogeneous spheroids were created and embedded in collagen as before, but with pre-staining of CAF mitochondria with MitoTracker Red (MT Red) and MDA-MB-231 cells with CellTracker Green (CT Green). After 48 h, transferred mitochondria were seen in numerous MDA-MB-231 cells, indicating that the cargo is, at least in part, mitochondrial content (Fig. 2B).

**Fig. 3.**
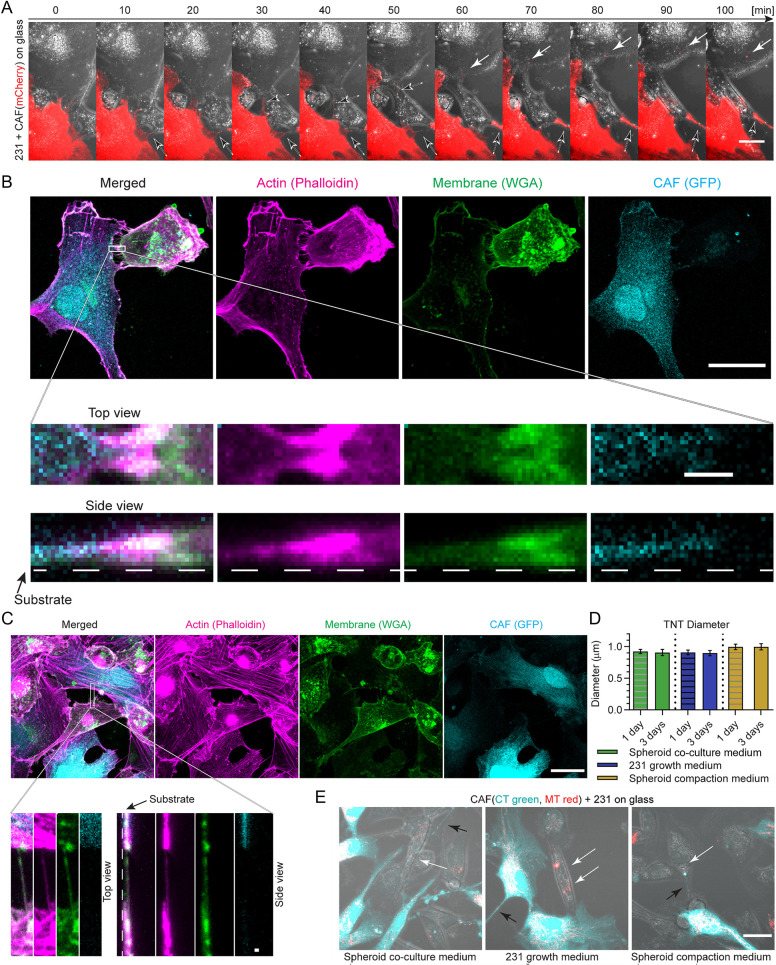
**CAF cargo transfer occurs via TNT formation.** (A) Representative time-lapse images showing TNT formation (black arrows) and the subsequent transfer of CAF cargo (white arrows) during 2D-co-culture of unlabeled MDA-MB-231 and CAF01-mCherry cells. Scale bar: 25 µm. (B,C) Representative images of TNTs formed between a CAF01-Casp9-GFP cell (cyan) and an MDA-MB-231 cell (unlabeled) on glass, with WGA-labeled cell membrane (green) and phalloidin-labeled actin filaments (magenta). Scale bars: 25 µm. Top views (horizontal plane) and side views (vertical cross-section) of boxed areas show a TNT formed from the CAF to the cancer cell (B) or from the cancer cell to the CAF (C), which is suspended above the glass substrate (as indicated by the dashed line) and contains actin filaments. Scale bars: 2 µm. (D) TNTs formed between CAF01 and MDA-MB-231 cells have an average diameter of 937 nm following 1-day co-culture when using spheroid co-culture medium, with no significant change in diameter seen at a later time (3 days). The average diameter did not vary significantly based on the medium composition (*n*=49, 23, 83, 28, 40, and 25, respectively). Error bars are mean±s.e.m. (E) Representative images of 2D co-culture of MDA-MB-231 (unlabeled) and CellTracker- and MitoTracker-labeled CAF01 (CT Green and MT Red) seeded at a 2:1 ratio following 2 days of co-culture in spheroid co-culture medium (left; containing 0.5% FBS), MDA-MB-231 growth medium (middle; containing 10% FBS), and spheroid compaction medium (right; containing 5% horse serum). White arrows indicate transferred mitochondria. Black arrows indicate TNTs. Scale bar: 25 µm. All data pooled from a minimum of three independent experiments.

Second, we aimed to investigate the method of mitochondrial cargo transfer. To identify whether cargo transfer was observed via EV communication, MDA-MB-231 cells were modified to transiently express a fluorescently labeled early endosome marker (Rab5a–GFP) (Poteryaev et al., 2010). When these cells were co-cultured with CAFs in spheroids, overlap of transferred CAF cargo and MDA-MB-231 endosomes was minimally observed (Fig. 2C). Specifically, image analysis revealed that 17.1±2.2% (mean±s.e.m.) of MT Red pixels within MDA-MB-231 cells were colocalized with the endosome signal, demonstrating that cargo transfer was not occurring primarily via endocytic uptake (Fig. 2C). To investigate other mechanisms of cargo transfer, we co-cultured CAF01-mCherry with unlabeled MDA-MB-231 cells on two-dimensional (2D) surfaces (Fig. 3A; Movie 2). Formation of TNT-like structures and subsequent cargo transfer were observed between the cells (Fig. 3A–C). TNTs are 50 to 1500 nm diameter tubular structures allowing for cargo transfer between distant and neighboring cells, which are still undergoing characterization, particularly in the field of tumor biology (Austefjord et al., 2014; Lou et al., 2017). Repeatedly, we observed TNTs with an average diameter of 0.9 µm following 1-day co-culture, which did not vary significantly based on the composition of the medium (ranging from 0.5–10% FBS) or over time (Fig. 3D). Consistent with previous reports on TNT formation (Lou et al., 2012), the TNTs we observed were actin-rich non-adhesive membrane structures often hovering above the substrate (Fig. 3B,C). We observed transfer of CAF mitochondria (Movie 3) independent of medium composition (Fig. 3E) although TNT formation is thought to be increased in hyperglycemic low-serum conditions (Lou et al., 2012). Together, our data suggest that, given sufficient cell–cell contact, CAFs transfer mitochondria-containing cargo to cancer cells, likely through the formation of TNTs.

### Inhibition of TNT attenuates CAF promoted cancer cell migration

**Fig. 4.**
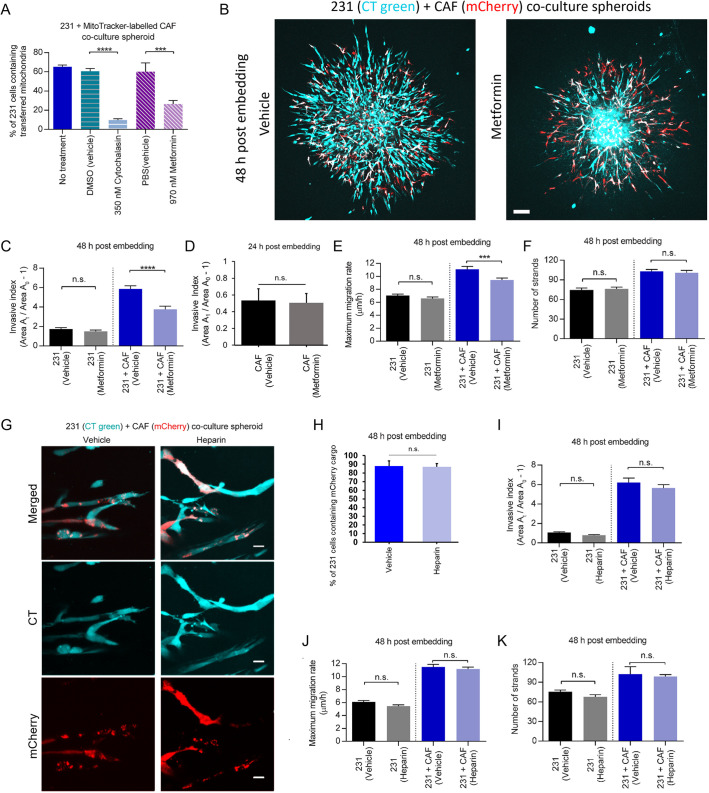
**Inhibition of TNT reduces CAF-promoted cancer cell migration.** (A) TNT blocking with cytochalasin B or metformin decreases the percentage of MDA-MB-231 cells containing transferred CAF mitochondria 24 h post embedding (*n*=357, 661, 650, 662 and 625 cells, respectively). (B) Representative images of co-culture spheroids of CellTracker-labeled MDA-MB-231 (CT Green) and MitoTracker-labeled CAF01 (MT Red) cells at 48 h post embedding given a vehicle control or metformin treatment. Scale bar: 100 µm. (C) Invasive index of mono-culture and co-culture spheroids 48 h post embedding and beginning treatment. (D) Invasive index of mono-culture CAF spheroids 24 h post embedding and beginning treatment (*n*=31 and 35 spheroids, respectively). (E) Maximum migration rate and (F) number of strands of mono-culture and co-culture spheroids 48 h post embedding. Blocking TNT formation with 970 nM metformin treatment (*n*=14, 22, 15, and 18 spheroids in C, *n*=14, 23, 18, and 19 spheroids in E, and *n*=14, 23, 16, and 19 spheroids in F, respectively) decreases invasive index and maximum migration rate, but does not affect the number of strand protrusions in co-culture spheroids. (G) Representative image of co-culture spheroids of CellTracker-labeled MDA-MB-231 (CT Green) and CAF01-mCherry cells with vehicle or 10 µg/ml heparin treatment 48 h post embedding and treatment. Scale bars: 20 µm. (H) Image analysis shows that heparin treatment does not affect the transfer of mCherry-containing cargo 48 h post embedding and treatment (*n*=33 and 61 cells across seven and nine independent 40× images, respectively). (I–K) Blocking of EV uptake with 10 µg/ml heparin does not affect the invasive index (*n*=24, 20, 14 and 20 spheroid in H, respectively), maximum migration rate (*n*=24, 20, 14, and 20 spheroids in I, respectively) and number of strand protrusions (*n*=19, 12, 9, and 19 spheroids, respectively) of mono-culture and co-culture spheroids 48 h post embedding and treatment. All data pooled from a minimum of three independent experiments. Error bars are mean±s.e.m. n.s., not significant (*P*>0.05); ***P<0.001, ****P<0.0001 [two-tailed unpaired Student's *t*-test (H), or one-way ANOVA followed by Sidak's multiple comparison testing (A,C,E,F,I–K)].

To investigate the role of cargo transfer via TNTs on MDA-MB-231 invasion, we used metformin or cytochalasin B to pharmacologically inhibit TNT formation without affecting endocytosis, as has been previously reported (Bukoreshtliev et al., 2009; Lou et al., 2012). Specifically, metformin decreases TNT formation by suppressing the mammalian target of rapamycin (mTOR) signaling pathway, which modulates TNT formation (Tossetta, 2022). Cytochalasin B blocks TNT formation owing to the selective elimination of filopodia (Bukoreshtliev et al., 2009). Treatments were given after spheroid formation, directly following collagen embedding. With both treatments, the percentage of MDA-MB-231 cells containing transferred CAF mitochondria decreased significantly in the co-culture spheroids (Fig. 4A; Fig. S3A). With metformin treatment, the invasive index and maximum migration rate of co-culture spheroids were dramatically reduced when compared to co-culture spheroids receiving the vehicle control (Fig. 4B–E). Metformin treatment did not affect the invasive index of MDA-MB-231- or CAF-only control spheroids (Fig. 4C,D). Cytochalasin treatment also significantly reduced the invasive index and maximum migration rate, but the observed effect was not isolated to TNT formation and subsequent cargo transfer, given that MDA-MB-231 mono-culture spheroids were also inhibited (Fig. S3B,C). Cytochalasin B, but not metformin, impacted the number of strands seen protruding from the co-culture spheroid (Fig. 4F; Fig. S3D), indicating that this might be a TNT-independent side effect resulting from the inhibited filopodia formation caused by cytochalasin B.

To further confirm that the cargo transfer promoted migration did not occur via EVs, EV uptake was inhibited through heparin treatment, which blocks proteoglycan surface receptors and promotes aggregation of EVs to decrease overall uptake (Atai et al., 2013; Christianson et al., 2013). To validate the effect of heparin on blocking EV uptake, EVs were isolated from CAF cells, size validated using a ZetaView (Fig. S3E), and given to collagen-embedded MDA-MB-231 mono-culture spheroids with or without heparin (Fig. S3F). Uptake of the exogenous EVs by MDA-MB-231 cells was seen to decrease significantly due to heparin when analyzed 10 h after the addition (Fig. S3G).

After validation was complete, heparin was introduced directly following collagen embedding of the co-culture spheroids, mirroring the other pharmacological inhibitor experiments. This blocking of EV uptake by heparin did not inhibit cargo transfer from CAFs to MDA-MB-231 cells in co-culture (Fig. 4G,H) and had no detectable impact on the migration of co-culture spheroids (Fig. 4I–K). Together, these data indicate that CAF-promoted migration can be attenuated by limiting mitochondrial transfer via TNTs, but not by limiting EV-based communication. This is especially interesting as heparin treatment here would block EV communication bi-directionally, indicating that neither breast cancer–CAF nor CAF–breast cancer EV communication is necessary for the CAF-induced increase in invasion observed in the co-culture spheroids.

### Presence of CAF during initial migration further enhances CAF-promoted migration

**Fig. 5.**
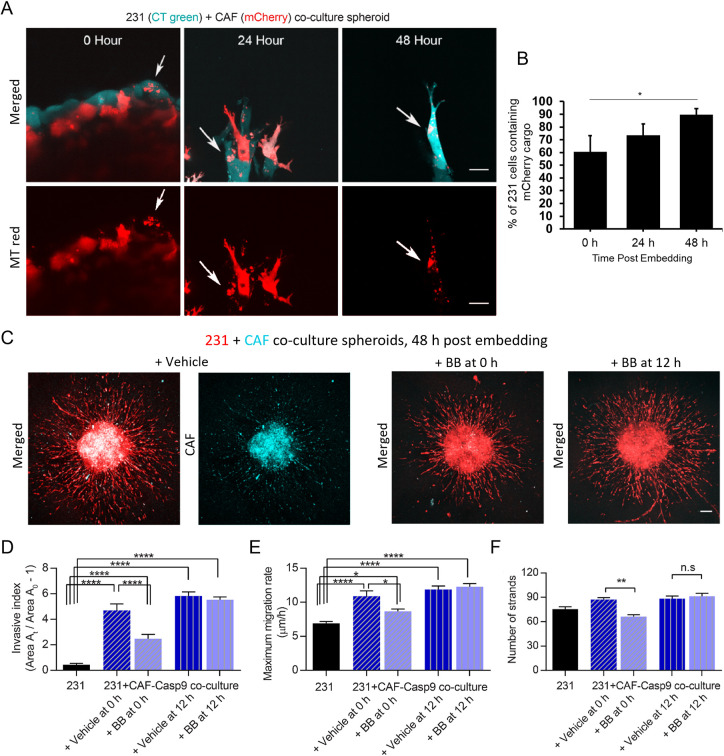
**Initial presence of CAFs during migration enhances migratory capabilities.** (A) Co-culture spheroids of CAF01-mCherry and CellTracker-labelled MDA-MB-231 cells (CT Green) at 0, 24 and 48 h post embedding (white arrows indicate transferred cargo from CAFs to cancer cells). Scale bars: 20 µm. (B) The number of MDA-MB-231 cells containing mCherry signal increases each day, with the increase being statistically significant between 0 h and 48 h (*n*=59, 38, 34 cells across 7, 7 and 11 independent images, respectively). (C) Representative images of co-culture spheroids of CellTracker-labeled MDA-MB-231 (red) and CAF01-Casp9-GFP (cyan) cells 48 h post embedding. Spheroids were treated with B/B homodimerizer to induce caspase-9 expression and apoptosis in CAFs at 0 h or 12 h post embedding or with vehicle control. Scale bar: 50 µm. (D,E) Induction of caspase-9 with the B/B homodimerizer at 12 h post embedding to kill the CAF01-Casp9 cells in the co-culture spheroid minimally affects the invasive index (D; *n*=17, 8, 17, 16 and 21 spheroids, respectively) and maximum migration rate (E; *n*=16, 10, 14, 16, and 19 spheroids, respectively) measured at 48 h post embedding, whereas treating the co-culture spheroid with B/B immediately (0 h) post embedding decreases the CAF-promoted cancer cell migration. (F) Induction of caspase-9 at 12 h post embedding to kill the CAF01-Casp9 cells in the co-culture spheroid minimally affects the number of strand protrusions (*n*=17, 10, 14, 16 and 19 spheroids, respectively) measured at 48 h post embedding, whereas treating the co-culture spheroid with B/B immediately (0 h) post embedding decreases the CAF-promoted increase in number of strand protrusions. All data pooled from a minimum of three independent experiments. Error bars are mean±s.e.m. n.s., not significant (*P*>0.05); *P<0.05; **P<0.01; ****P<0.0001 [one-way ANOVA followed by Sidak's (D–F) or Tukey's (B) multiple comparison testing].

Although our data suggest that CAFs transfer cargo to promote spheroid outgrowth, the requirement for the continued presence of CAFs following cargo transfer was unknown. Although the stromal and cancer cells were already in contact for 72 h to establish compact spheroids, we observed that the amount of transferred CAF cargo in cancer cells continued to increase post embedding in collagen (Fig. 5A,B). We aimed to test whether continued CAF presence, and therefore continued cargo transfer, was vital in the promotion of spheroid invasion. To do so, MDA-MB-231 cells were co-cultured in spheroids with CAFs expressing an inducible caspase-9 (CAF01-Casp9), which becomes active upon addition of an otherwise inert B/B homodimerizer to cause apoptosis of the CAFs (Fig. S4A) (Straathof et al., 2005). We induced caspase-9 expression in CAF01-Casp9 cells at different time points post embedding in collagen to kill the CAFs (Fig. 5C). Adding the B/B homodimerizer immediately post embedding caused a drop in the invasive index, maximum migration rate and number of strand protrusions when compared to values for the untreated co-culture spheroids (Fig. 5D–F). By contrast, adding B/B homodimerizer at 12 h post embedding did not cause a significant drop in any of these spheroid migration parameters, as compared to values in untreated co-culture spheroids (Fig. 5D–F). Together, our data indicate that the presence of CAFs directly post embedding is beneficial, but that the continued presence of CAFs, at least beyond 12 h, is not required for the enhanced invasion seen. Interestingly, even when CAFs were killed 0 h post embedding, the co-culture spheroids had significantly enhanced invasion as compared to for the mono-culture condition (Fig. 5D–F). These results were also confirmed using co-culture spheroids of SUM-159 and CAF01-Casp9 cells (Fig. S4B,C). This was likely due to the 3-day co-culture period prior to collagen embedding, which was necessary to allow compact spheroids to form. We therefore next investigated this pre-education period prior to collagen embedding to better isolate the effect of cargo transfer.

### CAFs educate cancer cells to promote migration through mitochondrial transfer

To further test whether the transferred cargo alone can promote cancer cell migration, we pre-conditioned or ‘educated’ breast cancer cells with CAFs using two different approaches. In the first approach, CAF01-mCherry and MDA-MB-231 cells were co-cultured in 2D on opposite sides of a 1-µm thick micro-porous membrane with >20% porosity (Fig. S5A), which allowed for TNT formation and cytoplasmic cargo exchange, as reported previously (Carter et al., 2017; Casillo et al., 2017). In the second approach, MDA-MB-231 cells were co-cultured in 2D with CAF01-Casp9 cells, which were subsequently killed. In each approach, after a 3-day education period, CAF-educated cancer cells were then used to generate mono-culture spheroids following the previously described 3-day compaction period followed by collagen embedding and imaging.

**Fig. 6.**
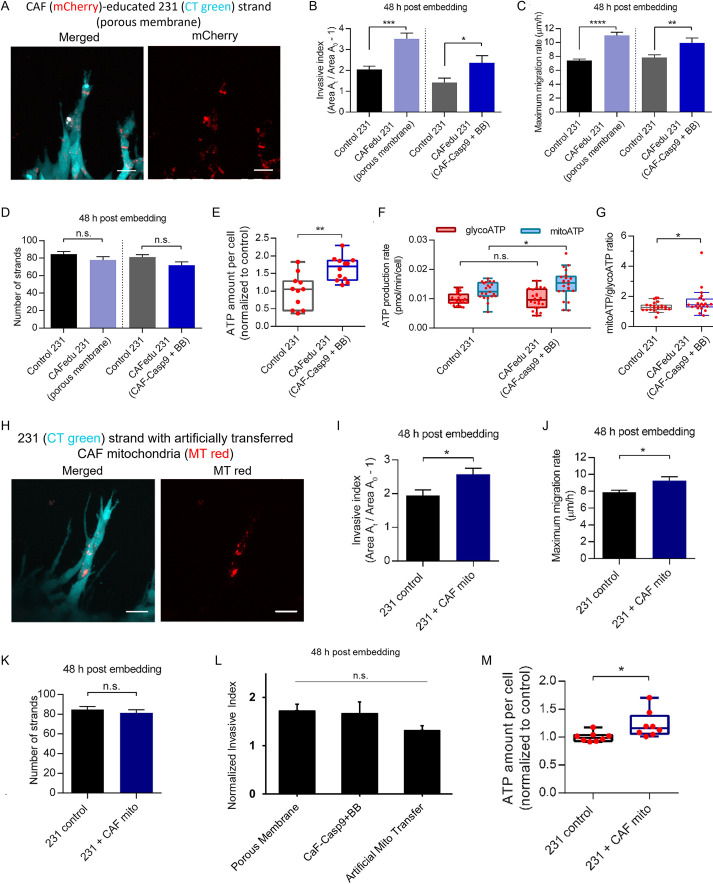
**CAFs educates cancer cells to increase migration via mitochondrial transfer.** (A) A representative image of CAF01-mCherry-educated migrating strand 24 h post embedding in collagen showing mCherry within CellTracker-labeled MDA-MB-231 (CT Green) cells. Scale bars: 20 µm. (B,C) CAF education of MDA-MB-231 cells (CAFedu 231) via micro-porous membrane co-culture (porous membrane) or co-culturing with CAF01-Casp9 followed by killing of CAFs with B/B homodimerizer-induced caspase-9 expression (CAF01-Casp9+BB) increases the invasive index (B; *n*=35, 15, 14 and 10, respectively) and maximum migration rate (C; *n*=31, 15, 14 and 10, respectively) of the spheroids made with CAF-educated cells as compared to those made with control MDA-MB-231 cells (control 231) 48 h post embedding. (D) CAF education (*n*=15, 24, 14 and 17 spheroids, respectively) does not affect the number of strand protrusions in MDA-MB-231 spheroids as compared to control at 48 h post embedding. (E) CAF education (*n*=12 wells) increases cellular total ATP level as compared to control (*n*=11 wells). (F) CAF education (*n*=22) increases mitochondrial ATP production (mitoATP) as compared to control (*n*=21) but minimally affects glycolytic ATP production (glycoATP). (G) CAF education increases the ratio of mitoATP to glycoATP as compared to control. (H) A representative image of an invading strand 24 h post embedding in collagen showing MitoTracker-labeled mitochondria (MT Red) artificially transferred from CAFs within CellTracker-labeled MDA-MB-231 cells (CT Green). Scale bars: 20 µm. (I,J) Artificial transfer of CAF mitochondria into MDA-MB-231 cells (231+CAF mito) increases the invasive index (I; *n*=20, and 24, respectively) and maximum migration rate (J; *n*=22, and 25, respectively) of spheroids 48 h post embedding as compared to control (231 control). (K) Artificial transfer of CAF mitochondria (*n*=24 and 25 spheroids, respectively) does not affect the number of strand protrusions in MDA-MB-231 spheroids as compared to control at 48 h post embedding. (L) Normalized to the control MDA-MB-231 cells for each experiment (value of 1), the invasive index is not statistically different between either pre-education method or between MDA-MB-231 cells with artificially transferred CAF mitochondria (*n*=15, 16, 25 spheroids, respectively). (M) Artificially transferred CAF mitochondria (*n*=8 wells) increases cellular total ATP level as compared to control (*n*=8 wells). All data pooled from a minimum of three independent experiments. Error bars are mean±s.e.m. Box-and-whisker plots are shown according to Tukey style. n.s., not significant (*P*>0.05); *P<0.05; **P<0.01; ***P<0.001; ****P<0.0001 [two-tailed unpaired Student's *t*-test (Welch corrected; E–G,I–K,M) or one-way ANOVA followed by Tukey's multiple comparison testing (B–D,L)].

Transferred mCherry-containing CAF cargo was observed within the spheroids, confirming education of cancer cells by CAFs during the initial period of co-culture (Fig. 6A). Spheroids generated from CAF-educated MDA-MB-231 cells from both approaches exhibited a significant increase in the invasive index and maximum migration rate compared to spheroids containing control MDA-MB-231 cells, without creating a significant difference in the number of observed strand protrusions (Fig. S5B; Figs 6B–D and 5B–D). Similar results were observed when SUM-159 cells were educated by the CAF01-Casp9 cells (Fig. S5C–E). These data further confirm that education of breast cancer cells by CAFs through the transfer of cargo is sufficient to enhance migration without the continued presence of the CAFs.

Given that our data indicate that mitochondria are transferred from CAFs to cancer cells (Fig. 2B), and mitochondria are responsible for energy production that powers cell migration (Cunniff et al., 2016; Desai et al., 2013), we hypothesized that mitochondrial transfer may impart a change in energy production in cells to enhance their migration. To test this hypothesis, we measured the ATP levels using a luminescence assay and confirmed an increase in ATP concentration in MDA-MB-231 cells upon CAF education (Fig. 6E). To further elucidate the effect of CAF education and mitochondrial transfer on the bioenergetics of the recipient cancer cells, we performed a Seahorse extracellular flux assay to measure ATP production rate (Fig. S5F). Compared to the control condition (non-educated MDA-MB-231 cells), CAF education increased the mitochondrial ATP production rate but not the glycolytic ATP production rate in MDA-MB-231 cells (Fig. 6F), which resulted in an increase in the relative contribution of mitochondrial respiration to total energy production in the cells (Fig. 6G). Increased energy production and mitochondrial respiration have been previously associated with increased breast cancer cell migration and metastasis (Davis et al., 2020; LeBleu et al., 2014; Zhang et al., 2019).

To further confirm that the transfer of mitochondria is responsible for the promotion of cancer migration, CAF mitochondria were isolated and artificially transferred into cancer cells prior to spheroid generation using a previously reported method (Kim et al., 2018) (Fig. 6H). Spheroids consisting of MDA-MB-231 cells with artificially transferred CAF mitochondria (Fig. 6H; Fig. S5B) had a significant increase in both the invasive index and maximum migration rate when compared to control (Fig. 6I,J), confirming that the transferred mitochondria are in part responsible for this phenotype. Specifically, when normalized against the non-educated control MDA-MB-231 cells in each experiment, there was no statistical difference in invasive index between MDA-MB-231 cells pre-educated by CAFs and those artificially given isolated CAF mitochondria (Fig. 6L). The number of strands was again unaffected (Fig. 6K). Similar to CAF-educated cells, MDA-MB-231 cells containing artificially transferred mitochondria had a significant increase in cellular ATP level when compared to control (Fig. 6M; Fig. S5G), confirming the role of artificially transferred CAF mitochondria in upregulating energy production in the recipient cancer cells. Together, these data indicate that CAFs educate breast cancer cells via mitochondrial transfer to promote their migration, likely through the upregulation of bioenergetics in cancer cells.

### Increasing mitochondrial ATP production with pyruvate inhibits invasion unless glycolysis is constant

**Fig. 7.**
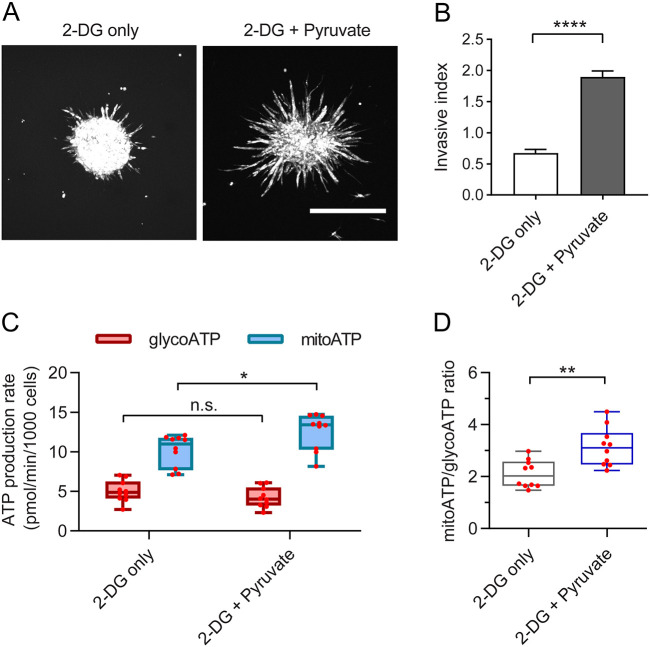
**Increased mitochondrial ATP production promotes cancer cell migration if glycolytic ATP does not reduce to compensate.** (A) Representative images of CellTracker-labeled MDA-MB-231 spheroids in collagen 48 h post embedding with or without 25 mM pyruvate supplementation, and with 25 mM 2-DG (2-DG+Pyruvate, 2-DG only, respectively). Scale bar: 500 µm. (B) In the presence of 25 mM 2-DG, 25 mM pyruvate significantly increases spheroid invasion (*n*=18 spheroids in each group). (C) In the presence of 25 mM 2-DG, 25 mM pyruvate (*n*=10; 2-DG+Pyruvate) increases mitochondrial ATP production (mitoATP) as compared to control (*n*=7; 2-DG only) but minimally affects glycolytic ATP production (glycoATP). (D) 25 mM pyruvate (*n*=10; 2-DG+Pyruvate) increases the ratio of mitoATP/glycoATP as compared to control (*n*=7; 2-DG only), in the presence of 25 mM 2-DG. All data pooled from a minimum of three independent experiments. Error bars are mean±s.e.m. Box-and-whisker plots are shown according to Tukey style. n.s., not significant (*P*>0.05); *P<0.05, **P<0.01, ****P<0.0001 (two-tailed unpaired Student's *t*-test).

Cancer cells are known to promote aerobic glycolysis in non-cancerous cells (such as CAFs) and use the metabolic waste products to increase their own OXPHOS production in a phenomenon known as the reverse Warburg effect (Shan et al., 2018). However, because the CAFs are removed after MDA-MB-231 pre-education and are not incorporated into the spheroid, we hypothesized that this reverse Warburg effect could not account for the increased OXPHOS and invasion seen after CAF pre-education (Fig. 6B–G). As a complementary approach to the pre-education, however, we next directly supplemented cell culture medium with different doses of pyruvate. Pyruvate is one of the main CAF byproducts of aerobic glycolysis associated with the reverse Warburg effect (Liang et al., 2022), and could therefore be used to investigate whether increased mitochondrial ATP production without CAF-mitochondrial transfer could impart a portion of the migratory advantage seen in the co-culture or pre-educated mono-culture spheroids. Surprisingly, the addition of pyruvate, especially at a high dose, significantly decreased spheroid invasion (Fig. S6A,B). Given that enhanced glycolysis is known to support cancer cell proliferation and migration (Hsu and Sabatini, 2008; Shiraishi et al., 2015), we speculated that the exogenous pyruvate was inhibiting glycolysis in a way not seen by mitochondrial transfer (Fig. 6F), which in turn caused a decrease in spheroid invasion. To test this, we measured mitochondrial and glycolytic ATP production rates in the presence of different doses of pyruvate using the Seahorse extracellular flux assay. Indeed, pyruvate supplementation increased mitochondrial ATP production at the cost of a decrease in glycolytic ATP production, such that the total ATP production rate was not significantly altered (Figs S6C and S7A). We therefore further hypothesized that increasing mitochondrial ATP production might promote cancer cell migration only if it does not inhibit glycolysis, but instead increases the total ATP produced. To test this hypothesis, we treated cells with 2-deoxy-D-glucose (2-DG) before adding pyruvate in the Seahorse assay. 2-DG suppresses glycolysis to the minimum baseline level required by the cell, by inhibiting hexokinase II, which catalyzes the initial metabolic step in the conversion of glucose (Pajak et al., 2019). Using 2-DG ensured that the addition of pyruvate could increase mitochondrial ATP production without simultaneously lowering glycolytic ATP production, such that the migration and metabolism could be further analyzed. 2-DG dramatically decreased glycolytic ATP production as expected (Figs S6F and S7B), and spheroid invasion was significantly inhibited by 2-DG (Fig. S6D,E). The addition of 2-DG slightly increased mitochondrial ATP (Fig. S6F), likely due to an attempt to increase total ATP production back to untreated levels. Compared to the condition with 2-DG alone, the addition of pyruvate at a high dose (25 mM) increased mitochondrial ATP production without further decreasing glycolysis (Fig. 7C,D; Figs S6F and S7B), which resulted in increased spheroid invasion as compared to the 2-DG-only condition (Fig. 7A,B). Given that mimicking the additional pyruvate availability associated with the reverse Warburg effect decreased the glycolytic ATP production rate and the invasion of breast cancer cells, this process alone is not sufficient to explain the increased invasion imparted by CAF inclusion in the tumor spheroids. Together, our data suggest that highly invasive cancer cells can further increase their migration by the acquisition of mitochondria from CAFs because this cargo transfer increases mitochondrial ATP production rates without altering glycolic ATP production (Fig. 6F,G), which increases total ATP in the cell (Fig. 6E,M).

## DISCUSSION

Metastatic solid tumor progression relies on the ability of malignant cells to migrate from the primary site. Cancer cell–stromal cell communication has been shown to impact tumor cell migration and can occur via numerous mechanisms, including through secretion of growth factors, chemokines and EVs (Donnarumma et al., 2017; Jin et al., 2017), as well as through altering the physical tumor microenvironment. In particular for physical manipulation, CAFs have been reported to promote the migration of less-invasive tumor cells by serving as a leader during collective migration via protease- and force-mediated matrix remodeling and microtrack formation (Gaggioli et al., 2007) and by cadherin-mediated mechanical force transmission (Labernadie et al., 2017). By contrast, more-invasive cancer cells, such as those with mesenchymal characteristics, have been shown to invade equally well by themselves without the presence of CAFs (Carey et al., 2013; Gaggioli et al., 2007).

We showed that the presence of patient-derived CAFs greatly enhanced the already highly invasive phenotype of MDA-MB-231 and SUM-159 breast cancer cells. However, this was particularly true across both CAF and breast cancer cell lines when the CAFs were co-cultured within the tumor spheroids, not when CAFs were embedded within the surrounding matrix of a mono-cultured breast cancer spheroid (Fig. 1; Fig. S1). In fact, regardless of whether CAFs were placed in the surrounding collagen or were part of the tumor spheroid, strands led by CAF cells were observed to be shorter than breast-cancer led strand protrusions (Fig. S2B). In co-cultured spheroids, however, less strands were led by CAFs, contributing to the increased invasion seen in this condition (Fig. S2A). Given that the breast cancer cell lines used can degrade and invade into collagen matrix at a relatively fast speed and that increased cellular contractility has been shown to reduce the speed of confined 3D cell migration (Mosier et al., 2019), it is possible that the highly contractile CAFs inhibited invasion when acting as the leader cell. Overall, the invasive index increased in co-culture spheroids, as compared to what was seen for the spheroids with CAFs surrounding a breast cancer mono-culture, because the length of the strands increased and because the total number of strand protrusions typically (but not always) increased with co-culturing (Fig. 1E–G; Fig. S1A). [The number of strands did not increase upon pre-educating the MDA-MB-231 cells (Fig. 6D) or by artificially transferring mitochondria (Fig. 6K)]. Additionally, the number of strand protrusions did not decrease upon inhibiting stromal cell–cancer cell communication through metformin or heparin (Fig. 4F,K). Instead, the presence of CAFs increased the invasive index due to an increase in the migration speed imparted after CAF cargo transfer via TNTs (Figs 4E and 5E), but not due to EV communication (Fig. 4J). In our experimental setup, only the initial presence of the CAFs was required to observe this change in migration speed and overall increase in invasion. Indeed, killing CAFs at 0 h post-embedding significantly reduced the invasive index, whereas killing CAFs at 12 h did not (Fig. 5D; Fig. S4B).

To further narrow down how CAFs pre-educate the breast cancer cells to increase their migratory ability without maintaining a continued presence of CAFs, we used different pre-conditioning approaches. The cargo transfer that occurred during the education period was sufficient to increase spheroid migration, although not to the same extent as when CAFs were present in the co-culture spheroid for at least 12 h post embedding (Figs 5D and 6B). This is likely due to, in part, that cargo transfer continued throughout the entire co-culture duration, with the percentage of MDA-MB-231 cells containing mCherry cargo increasing from ∼60% to 90% over the 48-h imaging period immediately following collagen embedding (Fig. 5B). Although the full contents of the cargo being transferred are not known and likely involve many components (de Rooij et al., 2017; Lou et al., 2012), mitochondria were observed within the transferred cargo. This alone could provide a benefit to the cancer cells, as changes in mitochondrial dynamics are known to regulate migration and directional persistence of invasive cancer cells (Desai et al., 2013; Zhao et al., 2013). This idea is supported by our previous report showing that an increase in ATP production by artificial mitochondrial transfer significantly increases the capability of MDA-MB-231 cells to lead and sustain strand invasion (Zhang et al., 2019). Additionally, we saw no significant difference in the migratory advantage imparted by CAF pre-education as compared to artificially transferring CAF mitochondria with no other direct CAF–cancer cell interactions (Fig. 5L).

We found that the mitochondria-containing cargo increased total and mitochondrial ATP production but minimally affected glycolytic ATP production. Increased mitochondrial OXPHOS has been linked to invasiveness and metastasis in breast cancer cells and poor patient survival (Davis et al., 2020; LeBleu et al., 2014). Leader lung cancer cells were also found to rely more on mitochondrial respiration during collective invasion (Commander et al., 2020). In 3D migration and invasion of single cells, mitochondria are transported toward the leading edge to provide localized ATP production (Cunniff et al., 2016; Kelley et al., 2019). However, enhanced glycolysis is also often positively correlated with 3D migration of breast cancer cells (Zanotelli et al., 2021). By measuring energy flux using the Seahorse assay after CAF pre-education, which we showed to substantially involve mitochondria transfer via TNT formation, and after pyruvate addition, we have begun to explore how the reverse Warburg effect and mitochondrial transfer might interplay in their effects. Specifically, when exogenous pyruvate was given, invasion was inhibited and glycolytic ATP was reduced to maintain a similar rate of total ATP production (Fig. 7C; Fig. S6A–C). As such, it is likely that there are additional signals and factors at play that allow cancer cells to utilize the pyruvate waste product from CAFs in the reverse Warburg effect to boost their total ATP production during specific periods of disease progression. One such factor might be the transferred mitochondria. Few papers have explored the metabolic effect of the recipient cell after mitochondria transfer via TNTs. The transfer of functional mitochondria in CAFs was shown to boost the oxidative metabolism of prostate cancer cells, but the effect on glycolysis for these cells was not investigated (Ippolito et al., 2019). Similar results were observed using acute myeloid leukemia cells co-cultured with bone marrow stromal cells in a previous study (Marlein et al., 2017). Another study by the same group reported an increase in OXPHOS with no change in glycolysis when two myeloma cell lines were cultured with bone marrow stem cells and mitochondrial transfer via TNTs was observed. (Marlein et al., 2019). This is similar to what we report here after CAF pre-education, although it is important to note that in the third myeloma cell line used, the increase in OXPHOS was coupled with a decrease in glycolysis after co-culture.

Additionally, restoring respiration is likely to affect more than just direct ATP generation. For example, DHODH-dependent respiration has been shown to be important for tumor formation but has little effect on ATP synthesis rates (Bajzikova et al., 2019; Liu et al., 2021). Cancer cells have also been reported to rescue themselves from chemotherapy-induced oxidative stress by acquiring mitochondria from CAFs, endothelial cells and immune cells via TNTs (Burt et al., 2019; Pasquier et al., 2013; Saha et al., 2022). TNTs can be initiated by cancer cells, and although mitochondrial transfer is sometimes reported to be bi-directional between cancer and other cell types, cancer cells are by far typically the recipient of the transfer (Zampieri et al., 2021). We observed only uni-directional transfer of the mitochondria and could not capture MT Red signal within CAFs at any timepoint (roughly 0, 12, 24 or 48 h) when MDA-MB-231 cells were stained with MT Red and CAF cells were stained with CT Green across 33 images taken in at least three separate experiments over a 2-month span (Fig. S4H). This uni-directional transfer of CAF mitochondria is not unique to our experiment or to breast cancer cells (Ippolito et al., 2019).

In conclusion, we demonstrate that contact-dependent TNT formation occurs between CAFs and breast cancer epithelial cells, and the transferred mitochondria-containing cargo increases the migratory ability of cancer cells. The artificial transfer of isolated CAF mitochondria was sufficient to induce this increase in cancer spheroid invasion to a similar level as pre-education of the breast cancer cells by CAFs through co-culturing. We further found that CAF pre-education of breast cancer cells promoted mitochondrial ATP production without inhibiting glycolysis, whereas delivery of exogenous pyruvate to breast cancer cells promoted mitochondrial ATP and inhibited glycolytic ATP levels. Additionally, we found that blocking TNT formation and subsequent mitochondrial transfer via metformin treatment attenuated the impact of CAFs on tumor cell migration, which emphasizes this interaction as a potential target against cancer migration and metastasis. Although specific inhibitors of TNT are not currently available, blocking the transfer or inactivating specific cargo might provide the same benefit in neutralizing CAF-induced migration (Burt et al., 2019). Taken together, our data suggest that there is an intricate communication between cancer and stromal cells which affects the metabolism of the former in a way unexplained solely by the reverse Warburg effect. However, much more work is needed to determine the mechanism by which transferred mitochondria alters recipient cell metabolism, as no study has yet delineated whether the transferred mitochondria are the direct cause of increased OXPHOS, or if there are intermediate steps in the cancer cells simply triggered by the transferred mitochondria. In particular, the transferred CAF mitochondria do not appear to retain the same canonical network structure once in the cancer cells. However, the mitochondrial network structure itself is much less interconnected in the MDA-MB-231 cells than in the CAF cells (Zhao et al., 2013), which might explain this difference, even if the CAF mitochondria are well integrated. One avenue of future work could include repeating the CAF pre-education and artificial mitochondrial transfer using CAF cells engineered with defects in their mitochondria, as well as performing super-resolution microscopy of the TNTs and resulting transferred cargo, such that the structure and function of said cargo could be further elucidated. Whether the metabolic and subsequent migratory effects are a direct result of the transferred functional mitochondria generating more ATP or an indirect result of the transferred cargo, CAF–cancer cell communication through TNTs appears to have a pronounced pro-tumorigenic impact on even aggressive breast cancer cells and this effect appears to continue beyond when the direct interaction has stopped.

## MATERIALS AND METHODS

### Cell culture and plasmids

MDA-MB-231 breast adenocarcinoma cells (American Type Culture Collection) were maintained in MDA-MB-231 growth medium containing high-glucose DMEM (4.5 g/l glucose; Life Technologies) supplemented with 10% fetal bovine serum (FBS; Atlanta Biologicals) (complete growth medium). SUM-159 breast carcinoma cells (BioIVT) were maintained in SUM-159 growth medium containing Ham's F-12 (Gibco), supplemented with 5% FBS, 10 mM HEPES (Gibco), 1 µg/ml hydrocortisone (Sigma-Aldrich) and 5 µg/ml insulin (Sigma-Aldrich). CellLight Early Endosomes–GFP (Invitrogen) was utilized to label early endosomes with GFP via transient transfection of a Rab5a and emGFP fusion construct following the manufacturer's instructions. Briefly, MDA-MB-231 cells were grown to 70% confluence, complete growth medium was removed and replaced with complete growth medium containing CellLight Early Endosomes-GFP (25 particles per cell). Cells were used for experiments following 16-h incubation with CellLight Early Endosomes–GFP.

Breast cancer-associated fibroblasts (CAFs) were isolated from remnant breast cancer tissues, after approval from the University of Alabama at Birmingham (UAB) Institutional Review Board for Human Use (IRB), in accordance with all IRB and institutional guidelines and regulations, including the Declaration of Helsinki, and after a waiver of patient authorization was requested and subsequently approved. Additionally, the CAFs were immortalized via transduction of human telomerase, all as previously described (Goliwas et al., 2016; Sadlonova et al., 2005). CAF01-hTERT and CAF32-hTERT (CAFs isolated from two different patients, referred to as CAF01 and CAF32 in the main text, respectively) were maintained in CAF growth medium containing low-glucose DMEM (1 g/l glucose; Life Technologies) supplemented with 10% FBS and 10 µg/ml Hygromycin (Mediatech). CAF01-hTERT-mCherry (referred to as CAF01-mCherry) were previously transduced to express mCherry as previously described (Goliwas et al., 2017), and were maintained in CAF growth medium supplemented with 2.5 µg/ml Puromycin (Mediatech). CAF01-hTERT were transduced to express an inducible caspase-9 (CAF01-hTERT-Casp9-GFP, referred to as CAF01-Casp9 or CAF01-Casp9-GFP) via pMSCV-F-del Casp9.IRES.GFP (Addgene plasmid #15567, deposited by David Spencer). Following viral transduction, fluorescence activated cell sorting (FACS) was completed to select for the GFP-positive cell population. CAF01-hTERT-Casp9-GFP cells were maintained in CAF growth medium.

All cell culture media are supplemented with 100 U/ml penicillin and 100 µg/ml streptomycin (Life Technologies). All cell culture, fluorescence imaging and time-lapse imaging were performed in a humidified environment at 37°C and 5% CO_2_. All cell lines used were tested for mycoplasma and deemed free of contamination.

### Induction of caspase-9 in CAFs

10 nM B/B homodimerizer (Clontech) was utilized to induce caspase-9 expression in CAF-hTERT-Casp9-GFP in co-culture experiments. 0.5 mM B/B homodimerizer stock (in ethanol, vehicle) was diluted to 10 nM in spheroid co-culture medium and spheroids were treated with B/B homodimerizer at 0 h or 12 h post collagen polymerization. For 2D pre-education experiments, caspase-9 induction in CAF01-hTERT-Casp9-GFP occurred following 3 days of co-culture. CAF-educated MDA-MB-231 or SUM-159 were isolated at 24 h following the addition of B/B homodimerizer and used to form mono-culture breast cancer spheroids.

### Cell and spheroid labeling

CellTracker (Green CMFDA or Orange CMRA, Invitrogen) was used to stain cell suspensions or spheroids, following the manufacturer's instructions. Briefly, cell suspensions were incubated in 10 µM CellTracker for 10 min at 37°C with 5% CO_2_ and washed in PBS or medium following incubation. Spheroids were incubated in 10–15 µM CellTracker for 20–30 min at 37°C with 5% CO_2_. MitoTracker Red CMXRos (Invitrogen) was used to stain mitochondria of attached cells 1 day prior to cell seeding by incubating cells in 50 nM MT Red for 30 min at 37°C with 5% CO_2_. Cells and spheroids were washed in PBS twice following the incubations.

### Multicellular spheroid generation and 3D culture

Multicellular spheroids were generated as previously described (Carey et al., 2013; Zhang et al., 2019). Briefly, cells were harvested and resuspended in spheroid compaction medium. Spheroid compaction medium was prepared by diluting Methocult (Stem Cell Technologies) to a final concentration of 0.25% in DMEM:F12 (Life Technologies) supplemented with 5% horse serum (Gibco), 20 ng/ml hEGF (Invitrogen), 100 ng/ml cholera toxin (Sigma-Aldrich), 0.5 µg/ml hydrocortisone (Sigma-Aldrich), 10 µg/ml insulin (Sigma-Aldrich) and 100 U/ml penicillin and 100 µg/ml streptomycin. 200 µl of cell suspension containing a total of 5000 MDA-MB-231 or SUM-159 cells (for mono-culture spheroids) or 200 µl of mixed cell suspension containing cancer cells and CAFs at a 2:1 ratio with a total of 5000 cells (for co-culture spheroids) was seeded into each well of non-adhesive round-bottom 96-well plate (Corning). Plates were centrifuged at 300 ***g*** for 5–10 min following seeding and then incubated at 37°C and 5% CO_2_.

After 3 days of compaction, spheroids were embedded in 4.5 mg/ml type I collagen gels with or without the presence of CAFs at 2.5×10^5^ cells/ml. Collagen gels were prepared as previously described (Carey et al., 2012). Briefly, type I collagen was acid-extracted from rat tail tendons (Rockland), purified via centrifugation and lyophilization, and reconstituted at 10 mg/ml in 0.1% acetic acid. The stock collagen solution was diluted to 4.5 mg/ml by gently mixing with ice-cold spheroid co-culture medium (MDA-MB-231 spheroid co-culture medium contains 0.5% FBS), and the solution was neutralized to pH 7.0 with 1 M NaOH. Spheroids were picked from 96-well plates and individually embedded within 200 µl collagen gels in glass-bottom 24-well plates (MatTek). After 30 min of gel polymerization at 37°C, gels were overlaid with 2 ml spheroid co-culture medium with or without pharmacological inhibitors, with medium change every other day.

### Microscopy and live-cell imaging

Static or time-lapse imaging were carried out with a Zeiss LSM800 confocal microscope, equipped with a temperature-, humidity-, and CO_2_-controlled environment chamber using the Zen software (Blue edition, v. Zen 2.3). Migration of tumor cells out of the spheroid core was monitored immediately following collagen polymerization and medium addition and at discrete time points over a 3-day period with a 10× NA 0.3 dry lens. Low-power *Z*-stack fluorescent images of spheroids were acquired from the bottom surface of the spheroid to the spheroid center and presented as maximum intensity *Z*-stack projections. Images were collected at least 200 µm above the bottom surface and edges of 3D matrices to avoid edge effects. Time-lapse images were acquired every 10 min for 3 h. High-resolution 2D confocal images and confocal reflectance images were acquired using a 40× NA 1.1 water-immersion lens.

### Migration analysis

Spheroid migration was calculated by measuring the projected spheroid area immediately after collagen embedding (*A*_0_) and the projected spheroid area following culture within collagen matrix (*A*_t_). The invasive index was defined as (*A*_t_/*A*_0_−1). Maximal migration distance was determined by measuring the radial distance from the spheroid edge to the migrating cell furthest from the spheroid. Maximum migration rate was determined by dividing the maximum migration distance by the time spent post spheroid embedding. To further investigate migration from co-culture spheroids, the number of migrating strands per spheroid was manually quantified over time using the maximum projection images and cell counter plugin in ImageJ.

### Analysis of collagen architecture

Cell localization and collagen fiber organization before and during spheroid migration were assessed with high-resolution confocal fluorescence and reflectance microscopy, respectively. Confocal reflectance images of collagen fibers are 1-μm thick confocal slices acquired as previously described near the spheroid periphery (Carey et al., 2012). The ImageJ plugin OrientationJ was used, as previously described, to measure collagen fiber orientation from confocal reflectance images (Rezakhaniha et al., 2012). To quantify collagen fiber organization, collagen fiber angle (θ_Fiber_) relative to the original spheroid surface (θ_Sph_) was determined for each pixel in the given region of interest, with the spheroid surface set to 0°. Using this analysis, the minimum angular difference was 0°, which corresponds to a tangentially aligned fiber; the maximum angular difference was ±90°, which corresponds to a radially aligned fiber. Results are graphed as distribution of orientation frequency and binned in 10° increments for all pixels within a region of interest.

### Evaluation of TNTs and mitochondrial transfer

The formation of TNTs was monitored in 2D co-culture using time-lapse confocal microscopy. Briefly, a 12-well glass bottom plate (Cellvis) was plasma treated for 3 min on high using an expanded plasma cleaner (Harrick Plasma) and UV treated for 1 h before plating 15,000 MDA-MB-231 cells in complete growth medium. Cells were allowed to adhere for ∼72 h before time-lapse imaging. At ∼24 h before imaging, MitoTracker Green (Invitrogen), reconstituted in DMSO, was diluted in low-glucose DMEM to a staining concentration of 12.5 nM. A T-25 flask of CAF01-hTERTs were rinsed twice with 5 ml of 1× PBS, incubated in the MitoTracker Green staining solution for 30 min, before being rinsed twice with 5 ml of 1X PBS, and cultured in CAF complete growth medium. At 24 h post staining, 20,000 MitoTracker Green-labeled CAF01-hTERTs were seeded onto the 12-well glass bottom plate wells containing MDA-MB-231s in MDA-MB-231 complete growth medium. The plate was equilibrated for 30 min in the LSM800 environment chamber before selecting positions and beginning imaging. The plate was imaged every 8.7 min for 6 h using a 20× NA 0.8 dry lens. 3D co-culture spheroids were imaged with a 20× NA 0.8 dry lens, following the procedure of imaging migration in spheroids. The number of cells per field containing transferred mitochondria were counted manually using ImageJ from both 2D and 3D conditions.

### Blocking TNTs and EVs

Metformin (Stem Cell Technologies) reconstituted in PBS at a final concentration of 970 nM (Lou et al., 2012) and cytochalasin B (Sigma-Aldrich) reconstituted in DMSO at a final concentration of 350 nM (Bukoreshtliev et al., 2009) were used to block TNT formation. To block EV uptake, heparin sodium (Sigma) reconstituted in H_2_O was used at a final concentration of 10 µg/ml (Christianson et al., 2013). Spheroid treatment was started immediately following the polymerization of collagen.

### CAF education of breast cancer cells

CAF education or pre-conditioning was completed by co-culturing MDA-MB-231 and CAF-hTERT-mCherry (2:1 ratio) on 1-µm thick microporous parylene membranes with 3-µm uniform pores (Carter et al., 2017; Casillo et al., 2017) for 3 days in spheroid co-culture medium. CAF-educated MDA-MB-231 cells were isolated from microporous membranes following the co-culture. Additionally, 2D co-culture of MDA-MB-231 and CAF-hTERT-Casp9-GFP (2:1 ratio) for 3 days in spheroid co-culture medium and subsequent induction of caspase-9 in CAFs with 10 nM B/B homodimerizer (24 h exposure) was utilized to isolate CAF01-Casp9-educated MDA-MB-231 cells. Cells were subsequently utilized for spheroid generation or measurement of ATP level and production.

### Isolation and artificial transfer of mitochondria

Mitochondria were isolated from donor CAFs by differential centrifugation and artificially transferred to the recipient MDA-MB-231 cells via centrifugation as previously described (Kim et al., 2018). In brief, CAFs were harvested, and cell pellets were homogenized using a disposable 1 ml syringe in SHE buffer containing 0.25 M sucrose, 20 mM HEPES pH 7.4, 2 mM EGTA, 10 mM KCl, 1.5 mM MgCl_2_, 0.1% bovine serum albumin (BSA), and 1× Halt protease inhibitor (Thermo Fisher Scientific), and centrifuged at 1100 ***g*** for 3 min at 4°C. The supernatant containing mitochondria was collected and centrifuged at 12,000 ***g*** for 15 min at 4°C to pellet mitochondria. The mitochondrial pellet was resuspended in 500 µl SHE buffer and centrifuged at 20,000 ***g*** for 10 min at 4°C. After removal of the supernatant, the pellet was resuspended in 50 µl PBS and kept on ice before use. Isolated mitochondria were quantified by determining the protein concentration using the DC protein assay (Bio-Rad). The recipient MDA-MB-231 cells were harvested, resuspended in PBS, and kept on ice before transfer. 1 to 5 µg of donor mitochondria per 1×10^5^ recipient cells was transferred to the recipient cell suspension, which was centrifuged at 1500 ***g*** for 5min at 4°C to finish the transfer. The recipient cells were then used for spheroid generation (containing 1 µg of donor mitochondria per 1×10^5^ recipient) or seeded for the ATPlite assay.

### Measurement of ATP concentration

Cellular ATP concentration was measured using the commercially available ATPlite luminescence assay kit (Perkin Elmer) following the manufacturer's instruction for MDA-MB-231 cells educated by CAF01-Casp9 cells or with artificially transferred CAF mitochondria. Briefly, the cells were seeded in 96-well plates with equal density and allowed to adhere and grow for 1 day in low-serum medium. The cells were then labeled with Hoechst 33342 and imaged to count cell numbers. Following cell counting, the cells were lysed with the lysis buffer and incubated with the luciferin-luciferase-based reaction buffer. The luminescent signal of each well was then detected using a microplate reader (BioTek Instruments). Following the ATPlite assay, the luminescent signals of each well were divided by the number of cells in each well and normalized to the average intensity of the control group.

### Seahorse ATP production rate measurement

When evaluating the effect of CAF education on cancer cell metabolism, CAF01-Casp9-educated and control MDA-MB-231 cells were seeded into Seahorse (Agilent) XFe96 microplates at 2×10^4^ cells per well, and the XFe96 cartridge were both hydrated overnight. The medium was replaced with the XF DMEM pH 7.4 with 10 mM of glucose, 1 mM of pyruvate, and 2 mM of glutamine and incubated at 37°C in a non-CO_2_ incubator for 1 h prior to the assay. The Seahorse real-time ATP rate assay was then carried out using the XFe96 analyzer following the manufacturer's instructions. 1.5 µM oligomycin and 0.5 µM rotenone and antimycin A were added sequentially to each well during the assay, with three readings made before, between and after the addition of the reagents.

When evaluating the effect of sodium pyruvate (Sigma) and 2-DG (Sigma) on cancer cell metabolism, MDA-MB-231 cells were seeded into a Seahorse XFe24 microplate at 5×10^4^ cells per well. The medium was replaced with XF DMEM pH 7.4 with 25 mM of glucose, and 4 mM of glutamine and incubated at a 37°C non-CO_2_ incubator for 1 h prior to the assay. The Seahorse real-time ATP rate assay was then carried out using the XFe24 Analyzer following manufacturer's instruction. 25 mM 2-DG, 0–25 mM pyruvate, 1.5 µM oligomycin and 0.5 µM rotenone (Rot) and antimycin A (AA) were added sequentially to each well during the assay, with three to eight readings made before, between and after the addition of the reagents.

The Wave software (Agilent) was used to measure the oxygen consumption rate (OCR) and extracellular acidification rate (ECAR) readings and the XF Real-Time ATP Rate Assay Report Generator (Agilent) was used to calculate the ATP production rate. Specifically, the ATP production rate is calculated from the OCR and ECAR readings using the following formulas according to the developer's manual:
(1)

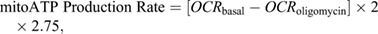

(2)

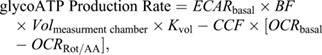
where *Vol*_measurement chamber_=5.65, and 2.28, *K_vol_*=1.19, and 1.60, CO_2_ Contribution Factor (CCF)=0.60, and 0.61, for Seahorse XFe24 and Seahorse XFe96 Analyzers, respectively; and Buffer Factor (BF)=2.5 for standard Seahorse assay medium. Units are not included in the above formulas.

Following the assay, the cells were fixed and stained with DAPI (Invitrogen) and imaged to count the cell number in each well. The final OCR, ECAR and ATP production rate values were normalized to the cell numbers.

### EV isolation and staining

EVs were isolated from CAF-mCherry cells using the ExoQuick^®^ ULTRA EV Isolation Kit for Tissue Culture Media (System Biosciences) following the manufacturer's protocol. Number and size distributions of the EVs were measured by nanoparticle tracking analysis with a Zetaview (Particle Metrix). Following isolation, EVs were stained with the FM 1-43FX fixable membrane stain (Invitrogen) and the ExoQuick^®^ ULTRA EV Isolation Kit for Tissue Culture Media was used to concentrate the EVs. EVs were resuspended in 250 µl spheroid co-culture medium. 100 µl of EV suspension was added to spheroid co-culture with and without heparin treatment.

### Fabrication of parylene membranes

Ultrathin parylene membranes were fabricated using common microfabrication techniques. 150 mm silicon wafers (University Wafers, MA, USA) were coated with 2% Micro-90 soap solution (Cole-Parmer, IL, USA) at 2000 rpm for 45 s, which serves as a water-soluble sacrificial layer. A target thickness of 800 nm parylene was deposited on the Micro-90-coated wafers using a PDS 2010 LABCOTER™ 2 Parylene Deposition System (Specialty Coating Systems, IN, USA). MicroPrime MP-P20 (Shin-Etsu MicroSi. Inc., AZ, USA) was used as an adhesion promoter between the parylene and photoresist layer. Hexagonally oriented micropores with 6 µm center-to-center spacing were patterned and etched using standard photolithographic methods as described previously (Carter et al., 2017). Deionized water was used to facilitate membrane lift-off and membranes were bonded to custom cut silicone gaskets using ozone bonding as described by previously (Mazzocchi et al., 2014). Parylene membrane thickness was measured using a Tencore P2 profilometer (KLA-Tencor, Milpitas, CA, USA).

### Fluorescent staining

Propidium Iodide (Thermo Fisher Scientific) and Hoechst 33342 (Invitrogen) were utilized per manufacturer's instructions to stain spheroids 6 h post embedding in collagen and B/B homodimerizer or vehicle addition. CAF01-Casp9-GFP and MDA-MB-231 cells co-cultured on 2D surfaces were labeled with Alexa Fluor 633-conjugated wheatgerm agglutinin (WGA; Life Technologies) to label the cell membrane before fixing with 3.7% formaldehyde (Sigma-Aldrich) in PBS. The cells were then permeabilized with 1% Triton X-100 in PBS and stained with Alexa Fluor 568-conjugated phalloidin (Invitrogen) for actin filaments.

### Statistics

All statistical analyses were performed using GraphPad Prism 6 or Microsoft Excel. Data are presented as dot, bar or line graphs expressed as mean±s.e.m. Two data sets were compared using two-tailed unpaired Student's *t*-tests. Three or more data sets were compared by one-way analysis of variance (ANOVA) followed by a Tukey's or Sidak's multiple comparison test. Box-and-whisker plots were presented in the Tukey style with the whiskers representing 1.5× IQR (the difference between the 25th and 75th percentiles), along with all data points.

## Supplementary Material

10.1242/joces.260419_sup1 Supplementary information
