# Supplementary material for: Mitochondrial transfer from cancer-associated fibroblasts increases migration in aggressive breast cancer
Source: J Cell Sci. 2023 Jul 28;136(14):jcs260419. doi: 10.1242/jcs.260419 (PMC10400000; doi:10.1242/jcs.260419)
Supplement: Supplementary information [file joces-136-260419-s1.pdf]

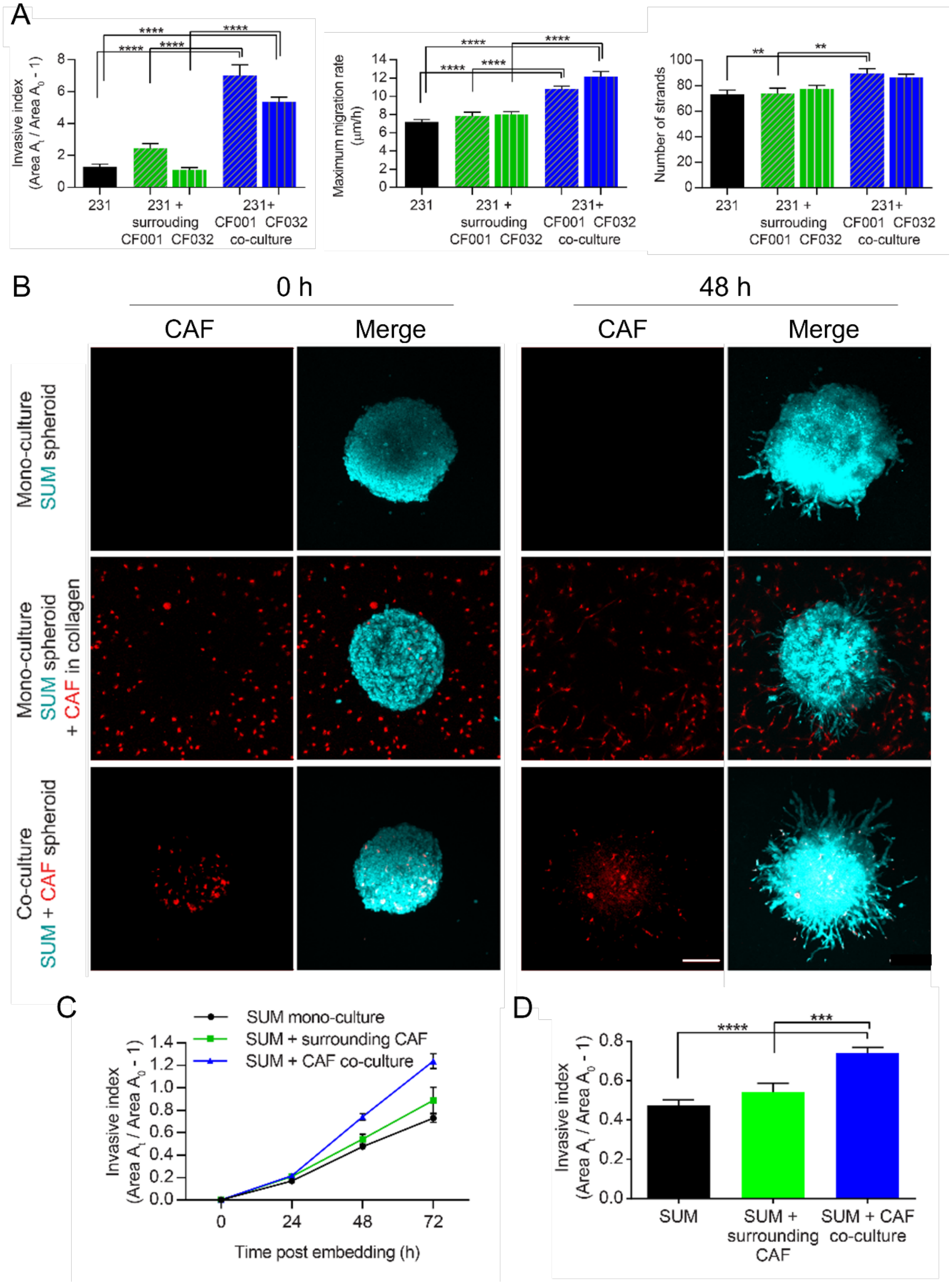

Journal of Cell Science • Supplementary information

Fig. S1. The promotion of breast cancer cell migration by CAFs derived from patients within co-culture spheroids. (A) CAFs from two different patients (CAF01, and CAF32) promote the migration of MDA-MB-231 cells when co-cultured within spheroids in terms of invasive index (N = 22, 17, 23, 28, and 21 spheroids, respectively) and maximum migration rate (N = 19, 16, 23, 28, and 21 spheroids, respectively) at 48 h post embedding. Number of strand protrusions at 48 hours post embedding is increased only with CAF01s in co-culture spheroids. **(B)** Representative images of a mono-culture CellTracker-labeled SUM-159 (cyan) spheroid alone (top) or with CAF01-mCherry (red) in the surrounding collagen matrix (middle), and a co-culture spheroid (CellTracker-labeled SUM-159 and CAF01-mCherry at 2:1 ratio; bottom) at 0 h (left) and 48 h (right) post embedding in 4.5 mg/ml collagen matrices. Scale bar, 200 µm. **(C)** Invasive index of mono-culture SUM-159 spheroids with or without surrounding CAF01-mCherry, and co-culture spheroids over 3 days. **(D)** Co-culture SUM and CAF spheroids (N = 55) have significantly increased invasive index at 48 h post embedding, as compared to mono-culture SUM-159 spheroids with (N = 31) or without (N = 31) surrounding CAF01-mCherry. Data pooled from a minimum of 3 independent experiments. Statistical significance assessed using one-way ANOVA followed by Sidak's multiple comparison testing (A, D). Error bars are mean ± S.E.M. ** $p < 0.01$, *** $p < 0.001$, **** $p < 0.0001$.

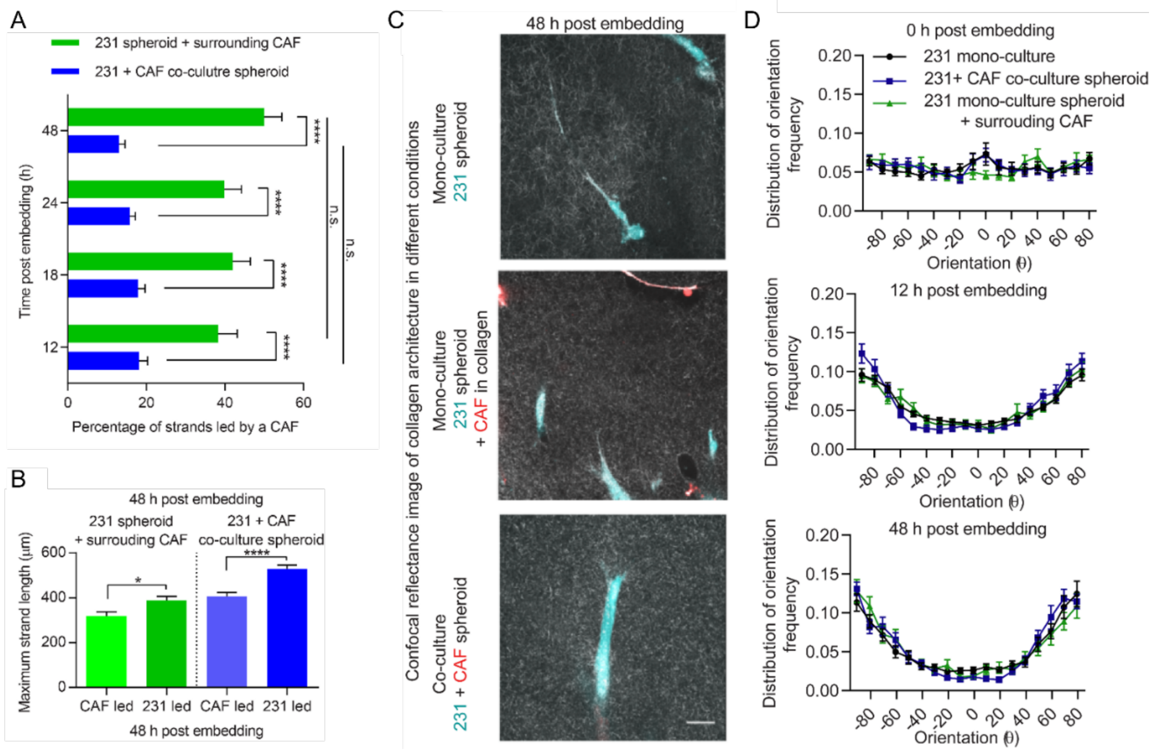

Fig. S2. The impact of CAF on tumor cell migration dynamics is not attributed to CAFs as leader cells or changes in the collagen architecture. (A) Percentage of MDA-MB-231 strands led by a CAF is significantly lower for the co-culture spheroid condition (N = 25 spheroids) than the mono-culture spheroid with surrounding CAF condition (N = 17 spheroids), while the percentages themselves do not change significantly over time. **(B)** The maximum length of CAF-led strands is shorter than MDA-MB-231-led strands in both mono-culture spheroids with CAFs in surrounding collagen (N = 17 spheroids) and co-culture spheroids (N = 30 spheroids) 48 h post embedding. **(C)** Representative confocal reflectance images of collagen architecture surrounding CellTracker-labeled MDA-MB-231 (cyan) mono-culture spheroids alone (top) or with CAF01-mCherry (red) in the surrounding collagen matrix (middle), and co-culture spheroids (bottom) at 48 h post embedding in 4.5 mg/ml collagen matrices. Scale bar, 20 µm. **(D)** Quantification of the collagen matrix orientation surrounding spheroids at 0 h (top), 12 h (middle), and 48 h (bottom) post embedding. An orientation angle of 0° indicates tangentially aligned fibers and ± 90° indicates radially aligned fibers relative to the spheroid edge. Data pooled from a minimum of 3 independent experiments. Statistical significance assessed using one-way (B) or two-way (A) ANOVA followed by Sidak’s multiple comparison testing. n.s. $p > 0.05$, *$p < 0.05$, **** $p < 0.0001$. Error bars are mean ± S.E.M.

Journal of Cell Science • Supplementary information

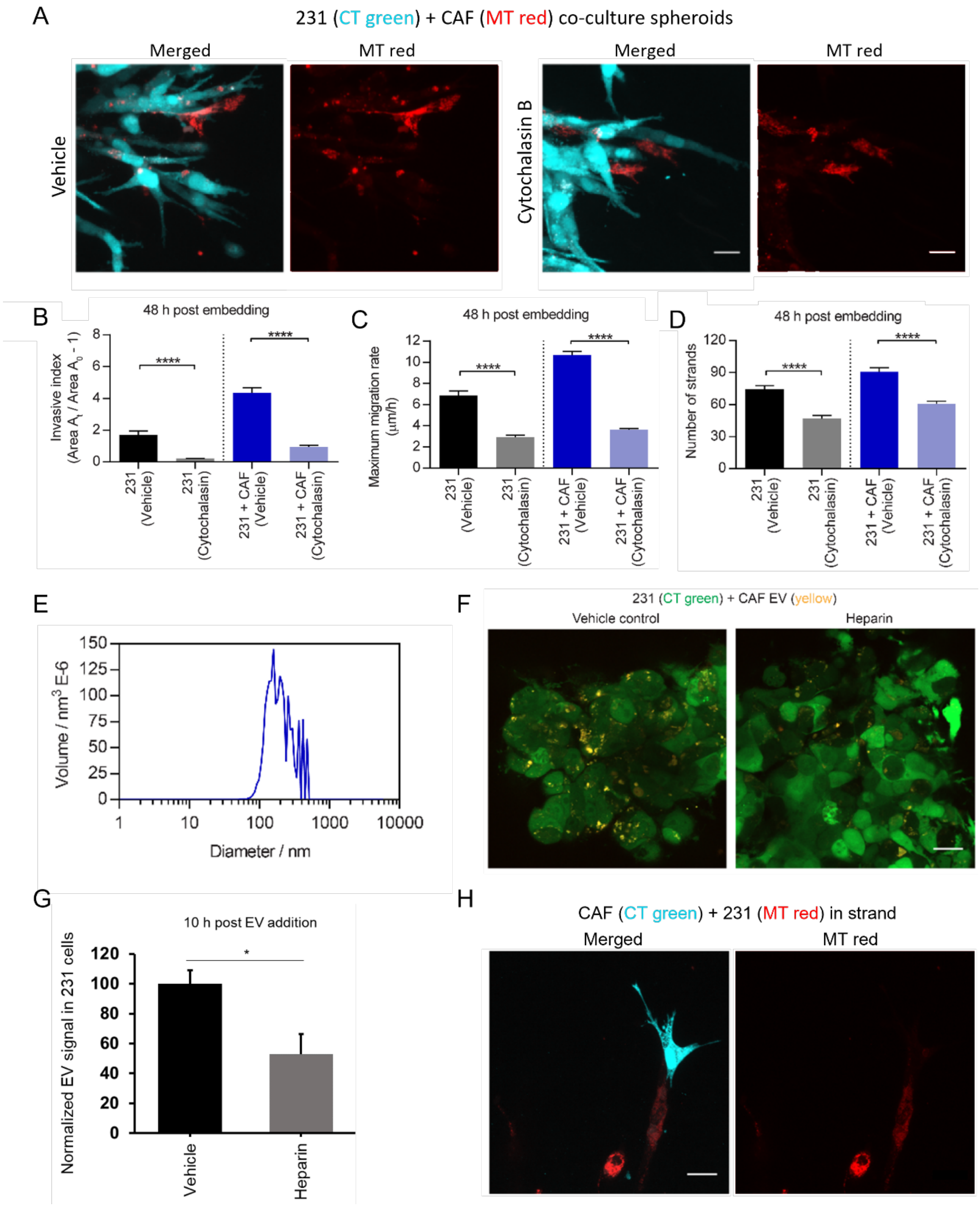

Fig. S3. Characterization of spheroid behavior with TNT or EV blockade. (A) Representative images of co-culture spheroids of CellTracker-labeled MDA-MB-231 (CT green) and MitoTracker-labeled CAF01 (MT red) showing decreased transferred

Journal of Cell Science • Supplementary information

mitochondria 48 h post embedding after 350 nM cytochalasin B treatment. Scale bar, 20 µm. **(B-D)** The invasive index, maximum migration rate, and number of strand protrusions 48 h post embedding after 350 nM cytochalasin B treatment (N = 24, 27, 25, and 38 spheroids in B, N = 26, 27, 28, and 38 spheroids in C, and N = 16, 18, 12, and 23 spheroids in D, respectively). **(E)** Nanoparticle analysis shows typical distribution of isolated EVs from CAF01-mCherry cells. **(F)** Representative images of CellTracker-labeled MDA-MB- 231 (CT green) mono-culture spheroids 10 h post embedding cultured with media containing isolated CAF EVs (yellow) and with 10 µg/ml heparin treatment or vehicle control. Scale bar, 20 µm. **(G)** Heparin treatment significantly blocked EV uptake in MDA-MB-231 cells (N = 5 and 6 independent images, respectively). Data normalized to the amount of EV signal seen in vehicle control as 100%. **(H)** A representative image of co-culture spheroid of MitoTracker-labeled MDA-MB-231 (MT red) and CellTracker-labeled CAF01 (CT green) within the collagen matrix at 48 h post embedding, showing no transfer of mitochondria from cancer cells to CAFs. Scale bar, 20 µm. Data pooled from a minimum of 3 independent experiments. Statistical significance assessed using Student's t-test (G) or one-way ANOVA followed by Sidak's multiple comparison testing (B-D). Error bars are mean ± S.E.M. * $p < 0.05$, **** $p < 0.0001$.

Journal of Cell Science • Supplementary information

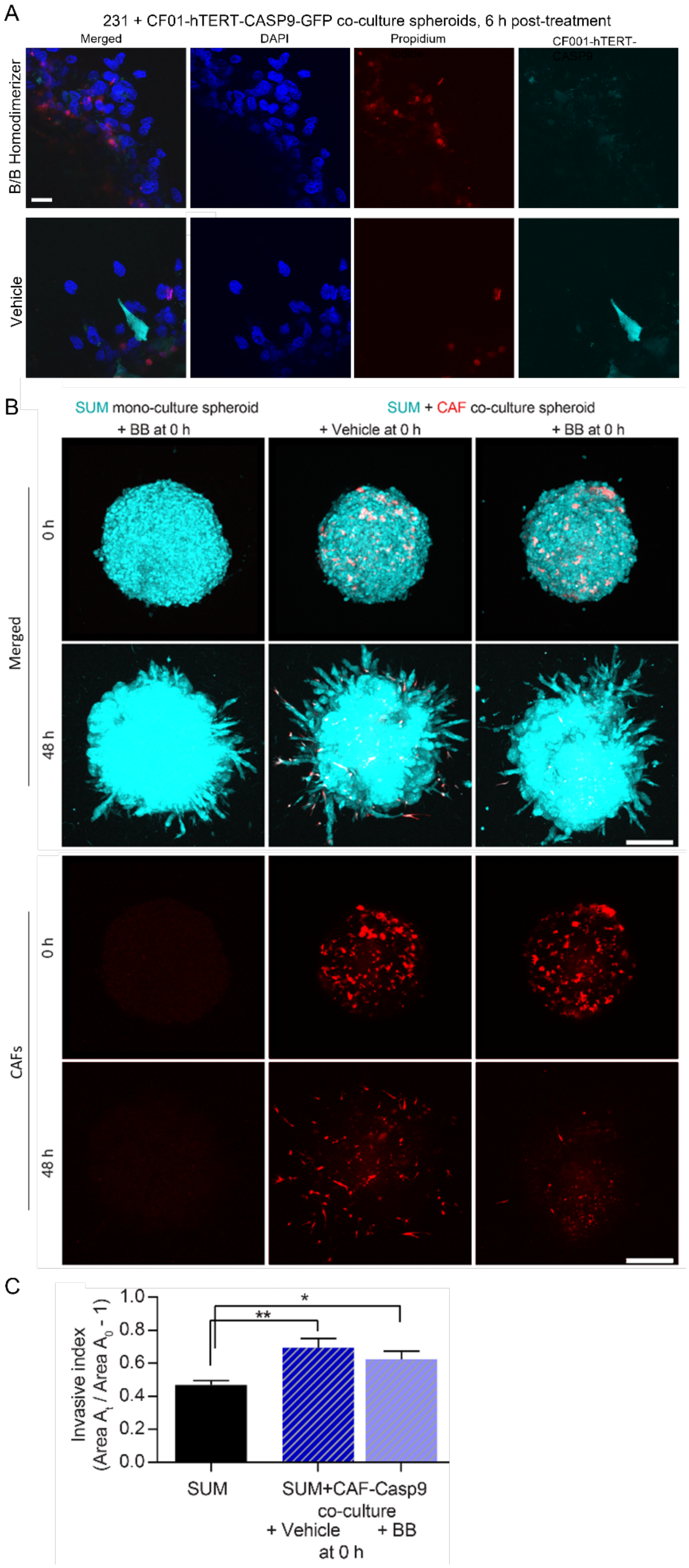

Journal of Cell Science • Supplementary information

Fig. S4. Impact of caspase-9 induction in CAFs on cancer spheroid migration. (A) Representative images of co-culture spheroids of MDA-MB-231 cells and CAF01-Casp9-GFP (cyan) 6 h post treatment with B/B homodimerizer (top) or with vehicle control (bottom) stained with DAPI (blue) and propidium iodide (red). Scale bar, 20 µm. **(B)** Representative images of mono-culture SUM-159 spheroids or co-culture spheroids of CellTracker-labeled SUM-159 (cyan) and CAF01-Casp9 (red) cells at 48 h post embedding with B/B homodimerizer treatment or with vehicle control at 0 h post embedding. Scale bar, 200 µm. **(C)** Invasive index of mono-culture SUM-159 spheroids (N = 30 spheroids) or co-culture spheroids of SUM-159 and CAF01-Casp9 cells (N = 15, and 23 spheroids, respectively) at 48 h post embedding with B/B treatment or with vehicle control at 0 h post embedding. Data pooled from a minimum of 3 independent experiments. Statistical significance assessed using one-way ANOVA followed by Sidak's multiple comparison testing (C). Error bars are mean ± S.E.M. * $p < 0.05$, ** $p < 0.01$.

Journal of Cell Science • Supplementary information

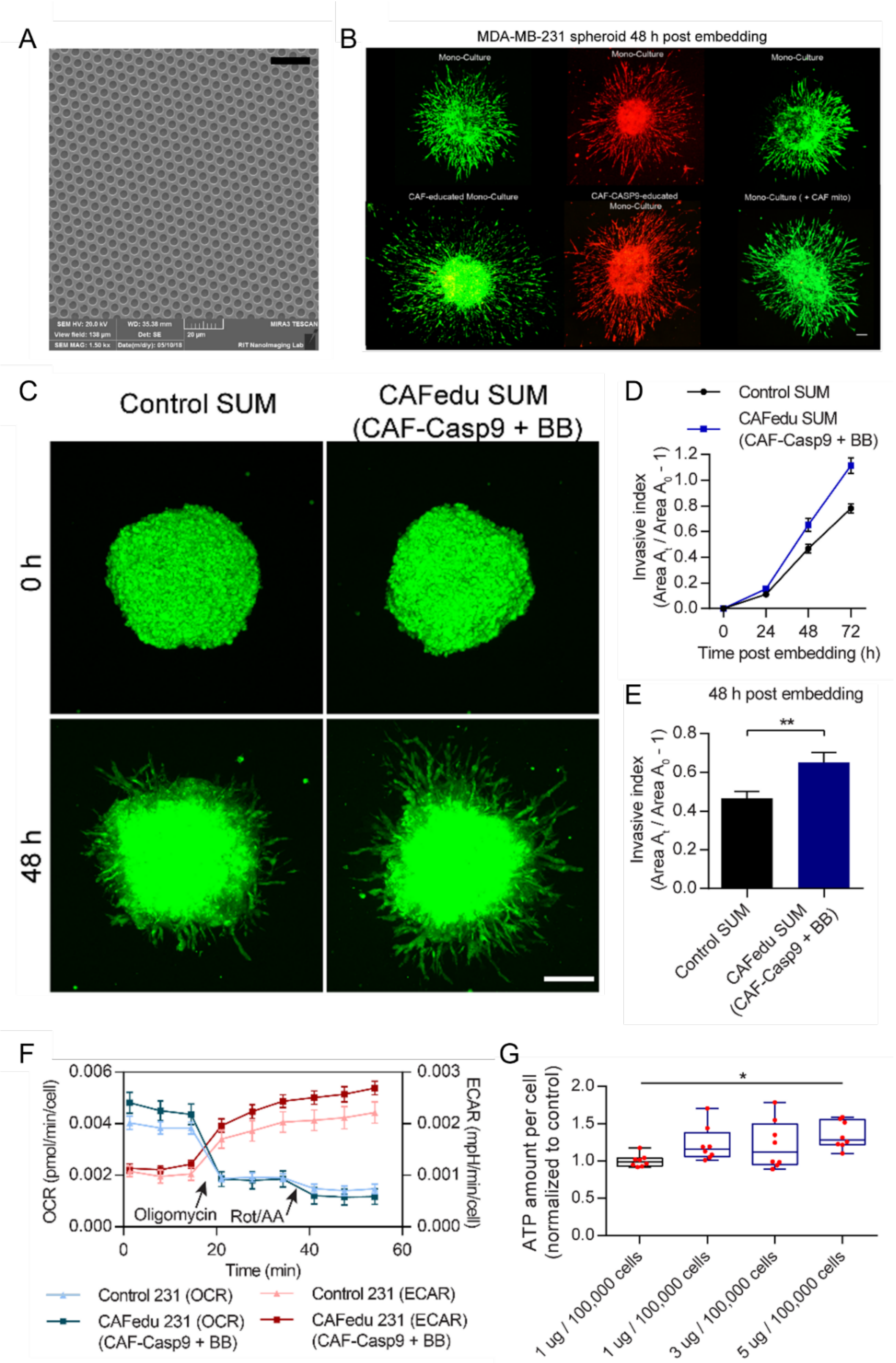

Journal of Cell Science • Supplementary information

Fig. S5. The effect of CAF education on breast cancer cell migration. (A) Scanning electron micrograph of the micropatterned ultrathin parylene membrane. Scale bar, 20 µm. **(B)** Representative images of CAF-educated CellTracker-labeled mono-culture MDA-MB-231 spheroids without pre-education (top row) or with pre-education (bottom) 48 h post embedding in 4.5 mg/mL collagen matrices. Education method is: Left, via co-culture on micro-porous membrane; Middle, via co-culture with CAF01-Casp9 followed with B/B treatment; Right, via artificially transferred CAF mitochondria). Scale bar, 20 µm. **(C)** Representative images of control and CAF01-Casp9-educated SUM-159 mono-culture spheroids at 0 h and 48 h post embedding in 4.5 mg/mL collagen matrices. **(D)** Invasive index of control and CAF-educated SUM-159 spheroids over 3 days. **(E)** CAF education increases the invasive index of SUM-159 spheroids at 48 h post embedding (N = 14, and 11 spheroids, respectively). **(F)** Dynamics of oxygen consumption rate (OCR; indicator of mitochondrial respiration) and extracellular acidification rate (ECAR; indicator of glycolysis) of CAF-educated (N = 22 wells) and control (N = 21 wells) MDA-MB-231 cells over the course of Seahorse real-time ATP rate measurement. Rot/AA, rotenone with antimycin A. **(G)** Cellular ATP level in MDA-MB-231 cells containing increasing concentrations of transferred CAF mitochondria (N = 8 wells for each group). Data pooled from a minimum of 3 independent experiments. Statistical significance assessed using Student's t-test (two-tailed; unpaired; E) or one-way ANOVA (G). Error bars are mean ± S.E.M. * $p < 0.06$. ** $p < 0.01$.

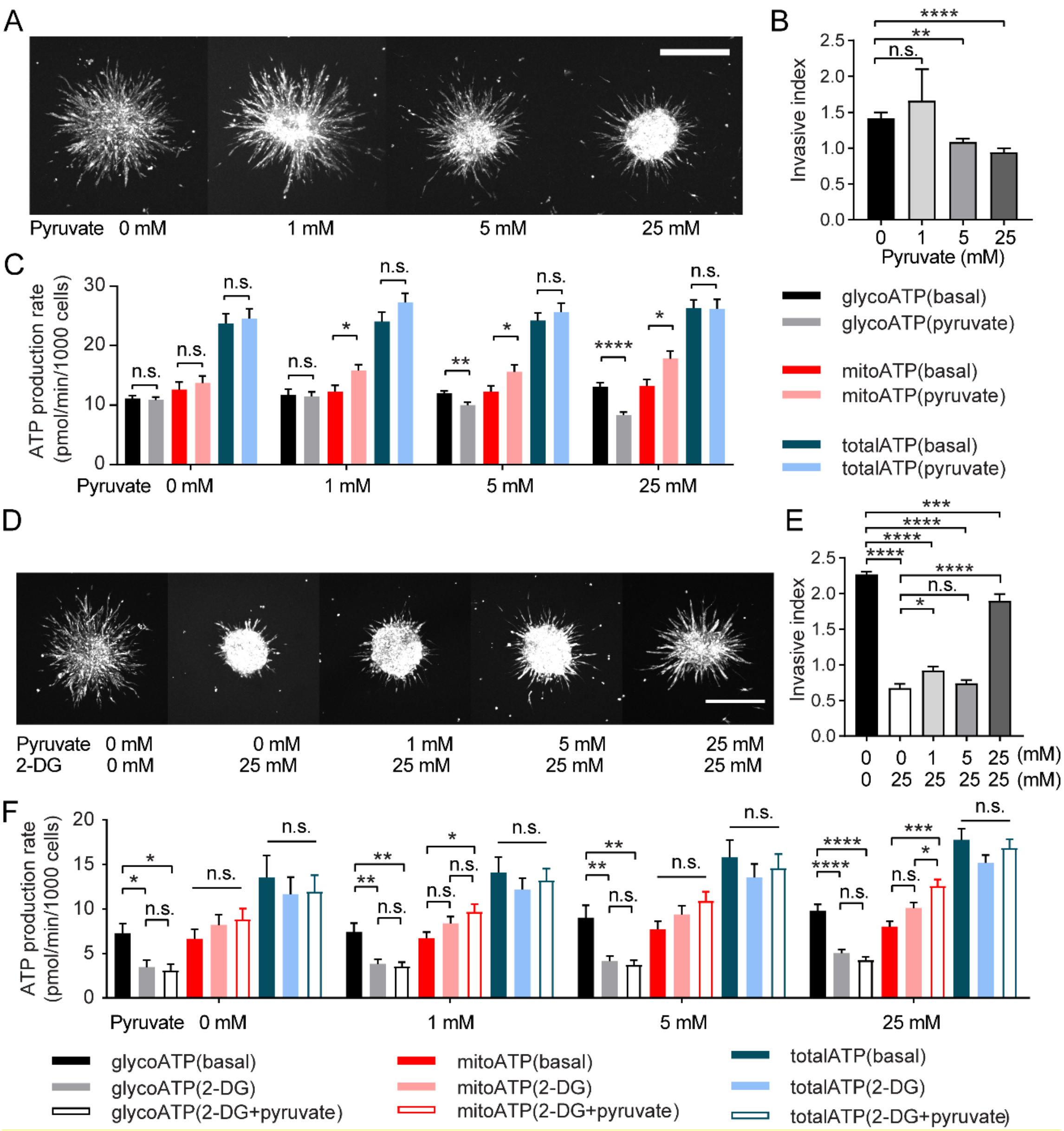

Fig. S6. The effect of increased mitochondrial ATP production on cancer cell migration depends on changes in glycolytic ATP production. (A) Representative images of CellTracker-labeled MDA-MB-231 spheroids in collagen 48 h post embedding with different doses of pyruvate supplementation. **(B)** Spheroid invasion at 48 h is significantly inhibited by high concentrations of pyruvate (N = 35, 20, 23, and 33, respectively). **(C)** Glycolytic, mitochondrial, and total ATP production rate (glycoATP, mitoATP, and

Journal of Cell Science • Supplementary information

totalATP= mitoATP + glycoATP, respectively) before and after the addition of pyruvate (basal and pyruvate, respectively) measured by Seahorse assay (N = 10 for each condition). **(D)** Representative images of CellTracker-labeled MDA-MB-231 spheroids in collagen 48 h post embedding with different doses of pyruvate supplementation, with or without 25 mM 2-DG. **(E)** Spheroid invasion at 48 h is significantly inhibited by 2-DG. With the presence of 2-DG, a high concentration of pyruvate significantly increases spheroid invasion (N = 23, 18, 24, 16, and 18, respectively). **(F)** Glycolytic, mitochondrial, and total ATP production rate before and after the addition 2-DG and pyruvate (basal, 2-DG, and 2-DG+pyruvate, respectively) measured by Seahorse assay (N = 7, 10, 9, and 10, respectively). Scale bar, 500 µm. Data pooled from a minimum of 3 independent experiments. Statistical significance assessed using one-way ANOVA followed by Sidak's multiple comparison testing (B, E, F) or Student's t-test (two-tailed, unpaired, Welch corrected; C). Error bars are mean ± S.E.M. n.s. $p > 0.05$, * $p < 0.05$, ** $p < 0.01$, *** $p < 0.001$, **** $p < 0.0001$.

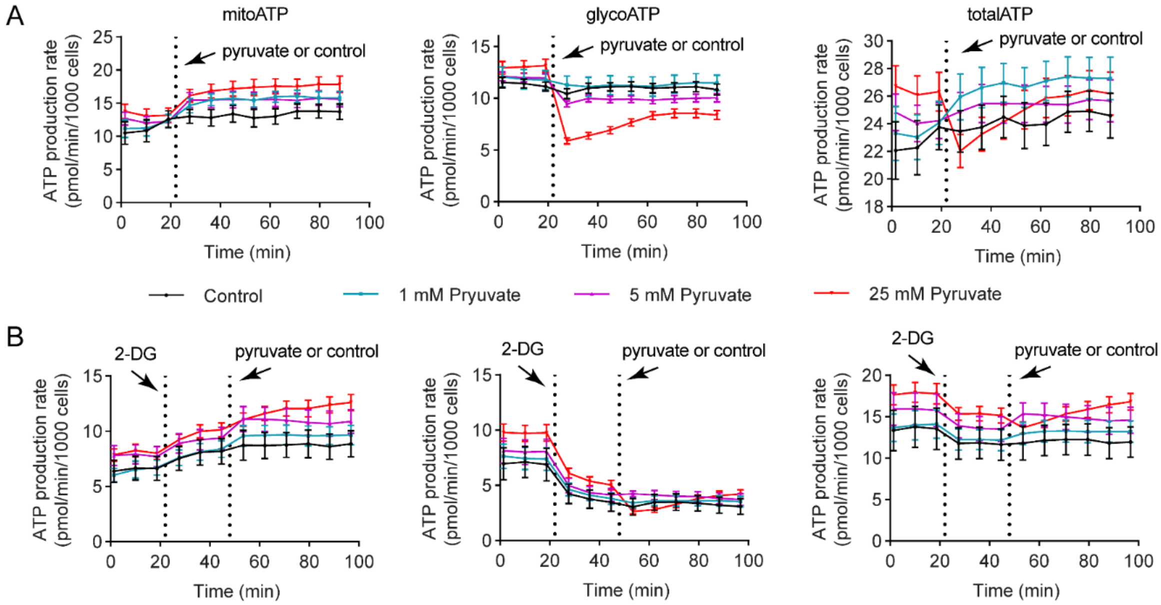

Fig. S7. Dynamics of ATP production rate in cancer cells upon 2-DG and pyruvate treatment. (A) Mitochondrial, glycolytic, and total ATP production rate (mitoATP, glycoATP, totalATP = mitoATP + glycoATP, respectively) as a function of time in MDA-MB-231 cells measured by Seahorse assay. 0-25 mM pyruvate is added at t = 21 min (N = 10 for each condition). **(B)** Mitochondrial, glycolytic, and total ATP production rate as a function time in MDA-MB-231 cells. 25 mM 2-DG is added at t = 21 min, 0-25 mM pyruvate is added at t = 45 min (N = 7, 10, 9, and 10, respectively). Data pooled from a minimum of 3 independent experiments. Error bars are mean ± S.E.M.

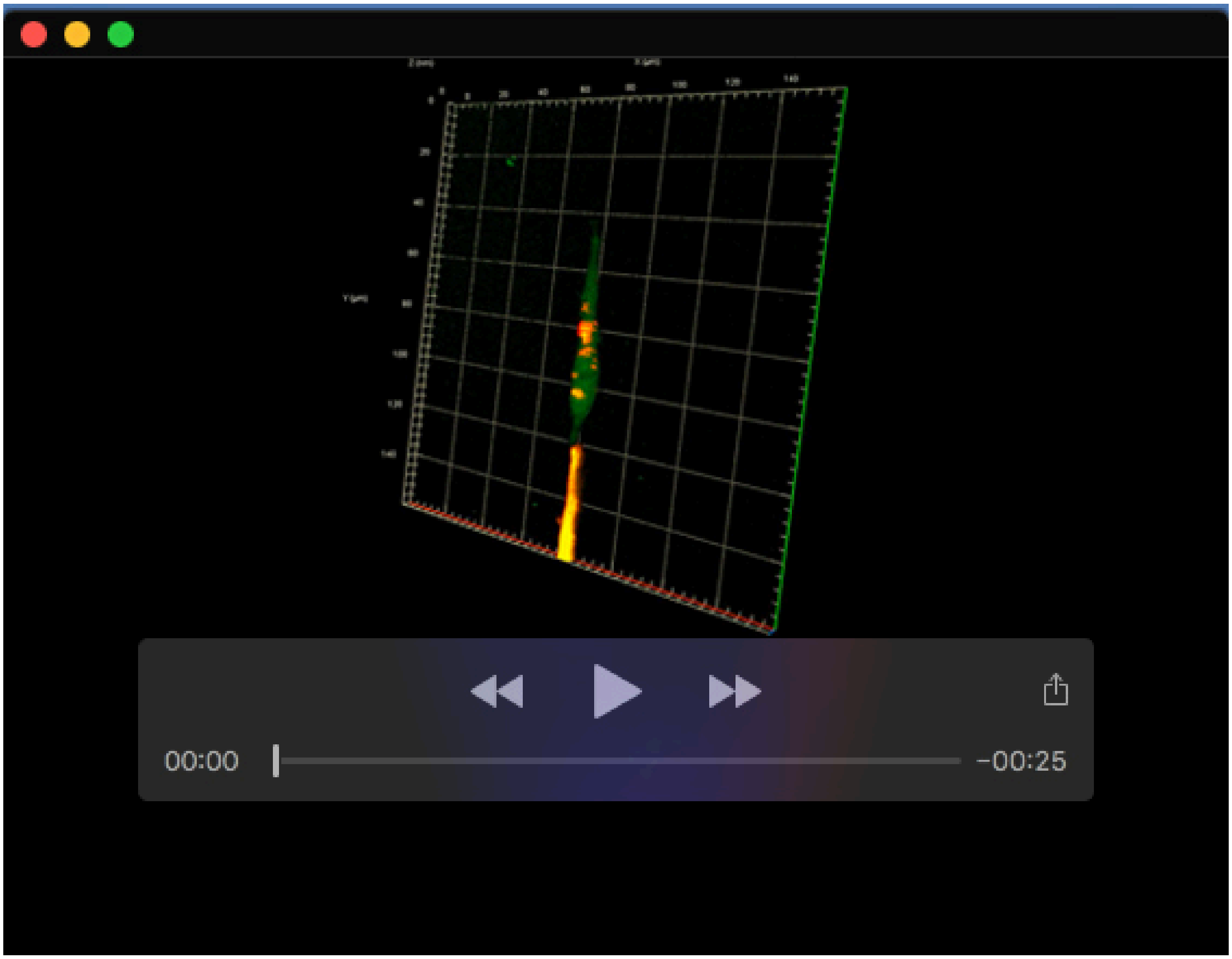

Movie 1. Representative 3D rendering of migrating strands of co-culture spheroids 48 h post embedding, with CellTracker-labeled MDA-MB-231 cells (green) containing transferred mCherry (red) transferred from CAF01-meCherry cells (red).

Journal of Cell Science • Supplementary information

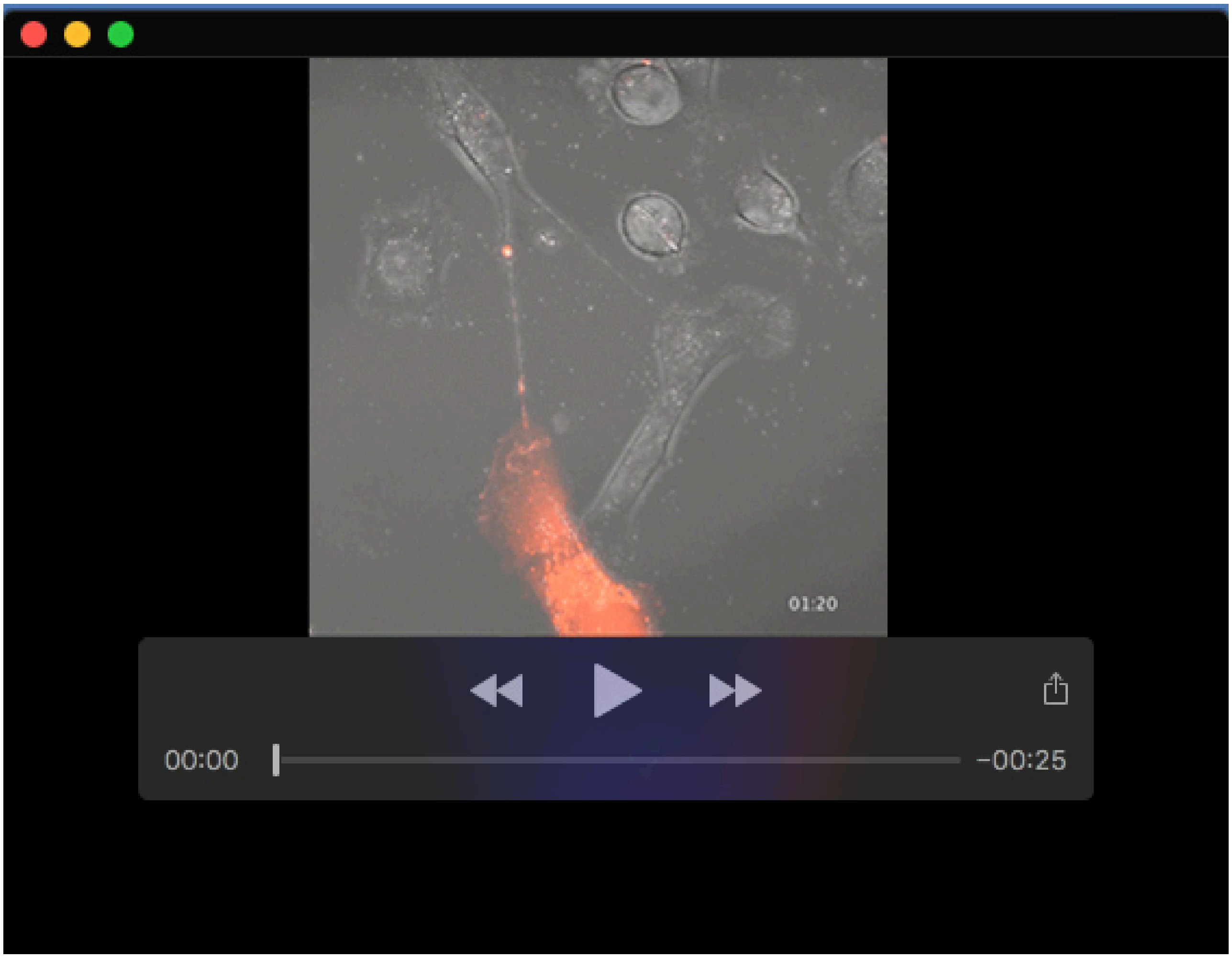

Movie 2. CAF transfer cargo to breast cancer cells via TNTs. Time lapse images over 3 hours showing CAF01-mCherry transferring cargo to unlabeled breast cancer epithelial cells. Time indicated in minutes.

Journal of Cell Science • Supplementary information

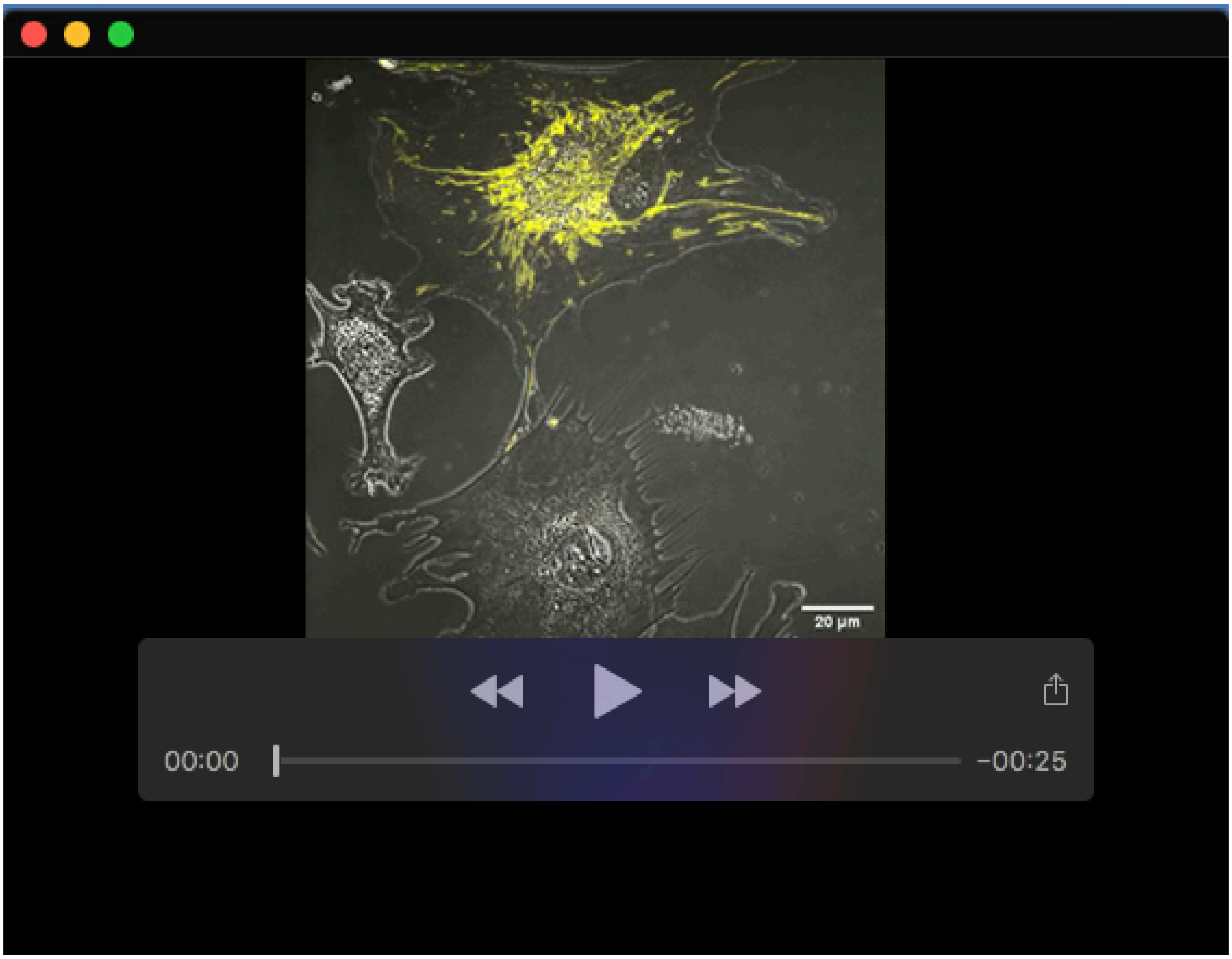

Movie 3. CAF transfer cargo to breast cancer cells via TNTs. Time lapse images over 6 hours showing MitoTracker Green-stained CAF01 cells transferring mitochondria (yellow) to unlabeled MDA-MB-231 cells.
